# Prediction of single-cell gene expression for transcription factor analysis

**DOI:** 10.1093/gigascience/giaa113

**Published:** 2020-10-30

**Authors:** Fatemeh Behjati Ardakani, Kathrin Kattler, Tobias Heinen, Florian Schmidt, David Feuerborn, Gilles Gasparoni, Konstantin Lepikhov, Patrick Nell, Jan Hengstler, Jörn Walter, Marcel H Schulz

**Affiliations:** Institute for Cardiovascular Regeneration, Goethe University, 60590 Frankfurt am Main, Germany; Theodor-Stern-Kai 7; Cluster of Excellence MMCI, Saarland University, Campus E1 7, Saarland Informatics Campus, 66123 Saarbrücken, Germany; Max Planck Institute for Informatics, Campus E1 4, Saarland Informatics Campus, 66123 Saarbrücken, Germany; Graduate School of Computer Science, Saarland University, Campus E1 3, Saarbrücken, Germany; Department of Genetics, Saarland University, Campus A2 4, 66123 Saarbrücken, Germany; Cluster of Excellence MMCI, Saarland University, Campus E1 7, Saarland Informatics Campus, 66123 Saarbrücken, Germany; Max Planck Institute for Informatics, Campus E1 4, Saarland Informatics Campus, 66123 Saarbrücken, Germany; Institute for Cardiovascular Regeneration, Goethe University, 60590 Frankfurt am Main, Germany; Theodor-Stern-Kai 7; Cluster of Excellence MMCI, Saarland University, Campus E1 7, Saarland Informatics Campus, 66123 Saarbrücken, Germany; Max Planck Institute for Informatics, Campus E1 4, Saarland Informatics Campus, 66123 Saarbrücken, Germany; Graduate School of Computer Science, Saarland University, Campus E1 3, Saarbrücken, Germany; Leibniz Research Centre for Working Environment and Human Factors (IfADo), Ardeystraße 67, 44139 Dortmund, Germany; Department of Genetics, Saarland University, Campus A2 4, 66123 Saarbrücken, Germany; Department of Genetics, Saarland University, Campus A2 4, 66123 Saarbrücken, Germany; Leibniz Research Centre for Working Environment and Human Factors (IfADo), Ardeystraße 67, 44139 Dortmund, Germany; Leibniz Research Centre for Working Environment and Human Factors (IfADo), Ardeystraße 67, 44139 Dortmund, Germany; Department of Genetics, Saarland University, Campus A2 4, 66123 Saarbrücken, Germany; Institute for Cardiovascular Regeneration, Goethe University, 60590 Frankfurt am Main, Germany; Theodor-Stern-Kai 7; Cluster of Excellence MMCI, Saarland University, Campus E1 7, Saarland Informatics Campus, 66123 Saarbrücken, Germany; Max Planck Institute for Informatics, Campus E1 4, Saarland Informatics Campus, 66123 Saarbrücken, Germany

## Abstract

**Background:**

Single-cell RNA sequencing is a powerful technology to discover new cell types and study biological processes in complex biological samples. A current challenge is to predict transcription factor (TF) regulation from single-cell RNA data.

**Results:**

Here, we propose a novel approach for predicting gene expression at the single-cell level using *cis*-regulatory motifs, as well as epigenetic features. We designed a tree-guided multi-task learning framework that considers each cell as a task. Through this framework we were able to explain the single-cell gene expression values using either TF binding affinities or TF ChIP-seq data measured at specific genomic regions. TFs identified using these models could be validated by the literature.

**Conclusion:**

Our proposed method allows us to identify distinct TFs that show cell type–specific regulation. This approach is not limited to TFs but can use any type of data that can potentially be used in explaining gene expression at the single-cell level to study factors that drive differentiation or show abnormal regulation in disease. The implementation of our workflow can be accessed under an MIT license via https://github.com/SchulzLab/Triangulate.

## Background

Single-cell sequencing has become a powerful tool to study gene expression patterns in different cellular contexts, such as cell differentiation, complex tissues, and disease. It is an open question how to best use single-cell RNA sequencing (scRNA-seq) data to infer cell-specific transcriptional regulatory programs.

Many methods have been developed that use gene expression data of pooled cell samples (bulk) to infer cell-specific transcription factor (TF) regulation. These methods often use the idea to decompose or associate variance in measured gene expression data with putative TF target gene sets to infer TF activity. To name a few examples, such approaches include network component analysis [1] and methods for predicting gene expression values from TF motifs  [2,3], combined with epigenetic [4] or chromatin conformation data [5, 6].

As scRNA-seq data protocols are becoming more widely adopted, novel methods have been developed that learn TF regulation by making use of the large number of cells obtained in current experiments (see overview [7]). For example the ACTION method [8] is an approach that identifies marker genes for each cell cluster from scRNA-seq data. It then uses a TF enrichment approach, using known TF-gene interactions, to determine TFs of regulatory importance for a set of marker genes in each cell cluster identified. Another approach, suggested by Ding et al. [9], uses a Kalman filter to model expression changes of single-cell clusters in differentiation processes by explicitly modelling the contribution of TFs in cell state transitions. scRNA-seq data were also used to build neuronal network classifiers that predict TF-gene target relationships by utilizing other types of information such as chromatin immunoprecipitation followed by sequencing (ChIP-seq) data [10].

SCENIC [11] is a widely used method for scRNA-seq data analysis. It uses a 3-step approach to infer regulatory networks. First, TF associations are inferred using regression trees that learn single-cell gene expression from the expression of TF-encoding genes. Second, these co-expression modules are tested for TF enrichment, such that significantly enriched TFs are used to define a TF regulon, by restricting to direct targets using motif information (window of 10 kb around the TSS or 500 bp upstream of the TSS). Third, the positively associated regulons are then incorporated with the single-cell data. Through this step, the activity of each regulon in each cell is evaluated by calculating an area under the curve (AUC) score, integrating the expression ranks across all genes in a regulon. Finally, these scores are used to create the desired activity matrix as output of their workflow. The authors mention that their approach could not find significant TF regulons where genes are negatively associated with TF expression, and thus the ranking in step 3 is limited to finding TF regulons among the highest expressed genes in a cell.

Later, Suo et al. [12] exploited SCENIC and modified it by defining a Jensen–Shannon divergence–based score to assess the cell type specificity of the regulons. By considering the regulons having high values of such a customized score, they were able to infer both known and novel regulatory elements in the mapped mouse cell atlas.

One of the appealing aspects of the SCENIC approach is that it is able to infer the TF activity per cell. However, because scRNA-seq data are noisy, this inference is challenging and, as stated above, negative associations cannot be made on a single-cell level in this way.

A widely adopted approach to overcome noise in challenging machine learning applications is the use of multi-tasking. In the context of bulk RNA-seq analysis several regression approaches that associate regulatory features with gene expression in a multi-tasking framework have been proposed [13–16].

In this work, we introduce TRIANGULATE, a tree-guided multi-tasking approach for inferring gene regulation in single cells. This work is conceptually similar to SCENIC [11] because it derives a TF activity score per cell, but it is methodologically different. Similar to SCENIC, we study the associations between single-cell gene expression and TFs. We train statistical models, where the expression measurements of genes across single cells are considered as the tasks in a multi-task learning (MTL) set-up. In contrast to SCENIC, we compute the binding affinities of many TFs instead of relying on the TF’s gene expression and explore the use of alternative ways for measuring TF activity, e.g., using bulk epigenetic data or TF ChIP-seq data of related cells.

We trained our models on 3 single-cell gene expression data sets, a data set comprising primary human hepatocytes (PHH) and *in vitro* differentiated hepatocyte-like cells (HLC), a data set of human skeletal muscle myoblasts (HSMMs), and the third a data set of normal and tumour samples from T cells of a patient with liver cancer. We inspected the coefficients of these models to identify interesting sets of features that best explain the gene expression in single cells. In addition, we compared the MTL results with standard univariate response regression models. These results indicate that the MTL models that integrate the information among all single-cell gene expressions not only produce more interpretable models but also often lead to higher accuracy.

## Materials and Methods

### Generating TF feature matrices

In this section, we explain how the feature and response matrices were generated for our statistical models. We define $F_{S}\in \mathbb {R}^{n\times p}$ to be the feature matrix representing the TF data measured for *n* genes, arranged at the rows, and *p* TFs, arranged at the columns. We generate the TF data in 3 different ways, as described below. In addition, we use the scRNA-seq data as the response variable for our statistical models. After applying the filtering steps described below, we apply a log_2_ transform to all feature and response matrices prior to the model-fitting phase.

#### Static features

TRAP [17] was run to quantify the binding affinities of 726 TFs at the promoter area defined by a window of size 2 kb centered at the transcription start site (TSS) using Position Weight Matrices from the TEPIC repository [18,19]. These affinity values form the “static" features.

#### Dynamic features

Using TEPIC version 2.0 [19], the binding affinities of 726 TFs were measured in peaks defined on the basis of the DNase I hypersensitive sites sequencing (DNase1-seq) data within the 50-kb window around the TSSs of HepG2 cells produced by DEEP [18], and mapped against human genome hg38. The contribution of TF motifs in DNase1-seq peaks are weighted using an exponential decay function in the 50-kb window as previously introduced [18]. In contrast to the static case, in this set-up, we additionally include 3 extra features representing the number of DNase peaks (Peak_Counts), the length of the open region (Peak_Length), and the aggregated DNase1-seq signal (Peak_Signal) computed within the 50-kb window around the TSS. A previous study showed that including these 3 features improves feature selection for gene expression prediction [4]. Because this particular type of feature is derived from the peaks in the DNase1-seq data that are able to capture the dynamics of DNA accessibility for TF binding, we refer to this set-up as “dynamic" features.

#### ChIP-seq features

ChIP-seq data for 123 TFs of the HepG2 cell line were downloaded from ENCODE, considering files processed by ENCODE's uniform processing pipeline. ChIP-seq read counts were measured in ChIP-seq peaks overlapping a 3-kb window defined around the gene’s TSS (mapped against genome hg38) to be combined with the HLC/PHH single-cell data for model training. We refer to these features as “ChIP-seq" features.

**Figure 1:**
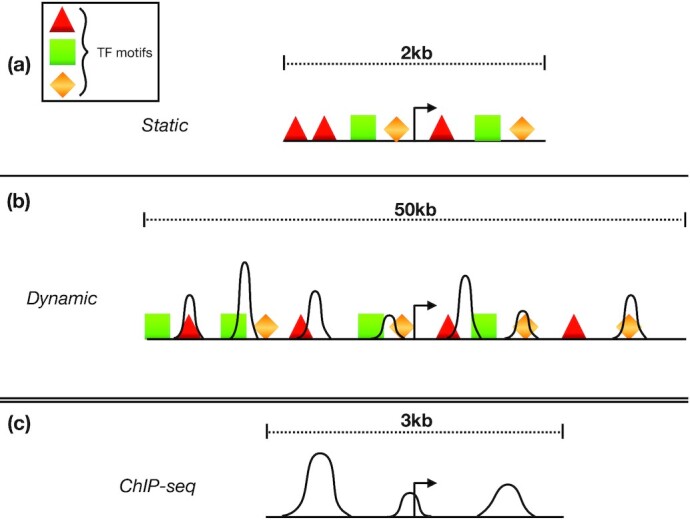
Genomic regions where the (a) static, (b) dynamic, and (c) ChIP-seq features are generated.

Fig. 1 illustrates the genomic region where the 3 feature set-ups (static, dynamic, and ChIP-seq) are generated.

#### Single-cell RNA-seq data as response for the statistical models

We generated scRNA-seq data for 657 HLCs differentiated from induced pluripotent stem cells/PHH (HLC/PHH) as described in the Supplementary Methods. These cells contain 2 annotated cell types, PHH and HLC, with 288 and 369 cells, respectively. Gene expression is quantified in transcripts per million (TPM). The expression values of all genes (TPM normalization) measured for a single cell are considered as a task for the MTL framework. The fastq files for this scRNA-seq data set have been submitted to the European Genome-phenome Archive (EGA) and are accessible via EGAS00001004201 accession code.

In addition, we obtained the HSMM data from [20]. It is worth mentioning that we only generated the static features for these data because there was no valid annotation of the cells that we could rely on for the downstream analysis in our study. Therefore, this data set was only used to demonstrate the results based on the different choices of tree structures required for the tree-guided MTL models.

#### Imputation of single-cell RNA-seq data

We used the scImpute method [21] on 2 scRNA-seq data sets. We set the parameter *k* to 2 for the HLC/PHH and 1 for the HSMM data. We use and compare the 2 variants of the gene expression data set, imputed and unimputed.

#### Filtering

We applied the filtering approach suggested by Monocle’s tutorial (Monocle2, RRID:SCR_016339) [22] on the scRNA-seq data. At first, the detected genes were defined using the detectGenes function by setting the min_expr argument to 0.1. A gene is kept if detected in ≥10 cells (based on the aforementioned definition of detected genes), otherwise discarded.

We further reduced the gene set by completely removing all the affinities computed for the genes for which the variance in their feature space (TF affinities) was less than the third quartile of the variances measured for each gene. More precisely, given the *F_S_* matrix, we compute the variance over the TF affinities for each genes, as follows:

(1) \begin{equation*} \mathrm{var}_i=\mathrm{variance}(F_{S}[i,]),\,\,i\in \lbrace 1,\cdots ,n\rbrace , \end{equation*}

where *F_S_*[*i*, ] is a vector of size *p*, holding the affinity values in the *i*th row. Next, we define a threshold *t* based on the third quartile computed over var_*i*_’s ∀*i* ∈ {1, ⋅⋅⋅, *n*}, as a cut-off to decide whether the gene_*i*_ should be kept:

(2) \begin{equation*} \mathrm{gene}_i: \left\lbrace \begin{array}{@{}l@{\quad }l@{}}\text{kept} & \text{if}\ \mathrm{var}_i \ge t\\ \text{discarded} & \text{else}\, . \end{array}\right. \forall i \in \lbrace 1,\cdots ,n\rbrace , \end{equation*}

In addition, we removed the TFs whose corresponding gene expression was zero.

Similarly, we applied these filtering steps on the other 2 feature set-ups, dynamic and ChIP-seq.

### Statistical learning frameworks

Here, we describe 2 distinct statistical learning frameworks, single-task learning (STL) and MTL. The MTL approach is further categorized into ordinary MTL (OMTL) and tree-guided MTL (TRIANGULATE).

We partitioned the data into training (60%) and test (40%) sets. Five-fold cross-validation was performed on the training set to select the best hyperparameters for all models. The TF and gene expression data are normalized to have zero mean and unit variance. We use Pearson correlation computed between the predicted expression and measured expression values on the same test set to assess performance for all models.

#### Single-task learning method

We trained individual regression models with elastic-net regularization through a 5-fold cross-validation model selection scheme, exploring the α parameter within the range of 0 and 1 with step size of 0.1 using the glmnet package in R [23].

#### Multi-task learning methods

Let $X\in \mathbb {R}^{n\times p}$ denote the input matrix for *n* observations (samples) and *p* features. Let $Y\in \mathbb {R}^{n\times k}$ denote the response matrix, whose columns are vectors of observations for *k* tasks. We look for an appropriate coefficient matrix $B\in \mathbb {R}^{p\times k}$ that establishes the linear relation between *X* and *Y* with the error term ϵ as described in the following formula:

(3) \begin{equation*} Y=XB+\epsilon \, . \end{equation*}

There are various ways to obtain the optimal values for the *B* coefficient matrix. In this section, we describe several MTL set-ups used in this study to understand the performance of different formulations and also downstream interpretation of the results.

#### Ordinary MTL

To optimize a multi-task regression model with elastic-net regularization, the following objective function is used:

(4) \begin{equation*} B^*=\mathrm{arg}\min _B \left[\Sigma _{i=1}^k(y_i - X\beta _i)^T.(y_i - X\beta _i) + \alpha \Sigma _{j=1}^p\Vert \beta ^j\Vert _2\right], \end{equation*}

where B* denotes the optimal coefficient matrix, α is a tuning parameter that controls the magnitude of the coefficients through the *L*_2_ norm regularization, and *y_i_* is a vector of size *n* holding the response values of the *i*th task. β_*i*_ denotes the coefficients corresponding to the *i*th task (column) of matrix *B*. Similarly, β^*j*^ denotes the *j*th row of matrix *B*.

Given the optimization formula, we trained an MTL model with elastic-net regularization using the R glmnet package [23], where the family argument was set to “mgaussian" to account for the multi-tasking nature of the set-up. We used 5-fold cross-validation to optimize over the α search grid defined within the range of 0 and 1 with the resolution of 0.05. The models generated using this formulation are hereinafter referred to as OMTL.

#### Tree-guided group-lasso MTL

In the ordinary MTL scenario all tasks share the same relevant features. However, it is possible that a subset of highly related tasks may share a common set of relevant features, whereas weakly related tasks are less likely to be affected by the same features. An improvement was proposed by Kim and Xing [24] to address this shortcoming of OMTL models. Through their proposed method, to which they refer as tree-guided MTL, the relationship among the tasks is represented as a tree *T* with *V* vertices. Each leaf node of *T* is associated with a task and the internal nodes reflect the groupings of the tasks. This tree structure can be inferred directly from the data or may be available as prior knowledge beforehand. Within this tree, each node *v* ∈ *V* is associated with a weight *w_v_*, typically representing the depth of the subtree rooted at node *v*. The optimization formula for tree-guided MTL is

(5) \begin{equation*} \begin{split} B^*=\mathrm{arg}\min _B [\Sigma _{i=1}^k(y_i - X\beta _i)^T.(y_i - X\beta _i)\\ + \lambda \Sigma _{j=1}^p\Sigma _{v\in V}\Vert w_v\beta ^{j}_{G_v}\Vert _2], \end{split} \end{equation*}

where λ is the regularization parameter and $\beta ^{j}_{G_v}$ is a group of regression coefficients $\lbrace \beta _i^j:i\in G_v\rbrace$. We used the LinearMTL package implemented in R [25] to train the tree-guided MTL models. We first partitioned 60% of the data for training and 40% for test. Then, we normalized the data to zero mean and unit variance. For the purpose of model selection, we performed a 5-fold cross-validation, through which 21 distinct values of λ, defined within the range of 0 and 1 with the resolution of 0.05, were explored. Finally, we trained the models by setting the maximum number of iterations to 1,000.

#### Construction of trees used for the tree-guided MTL models

**Figure 2:**
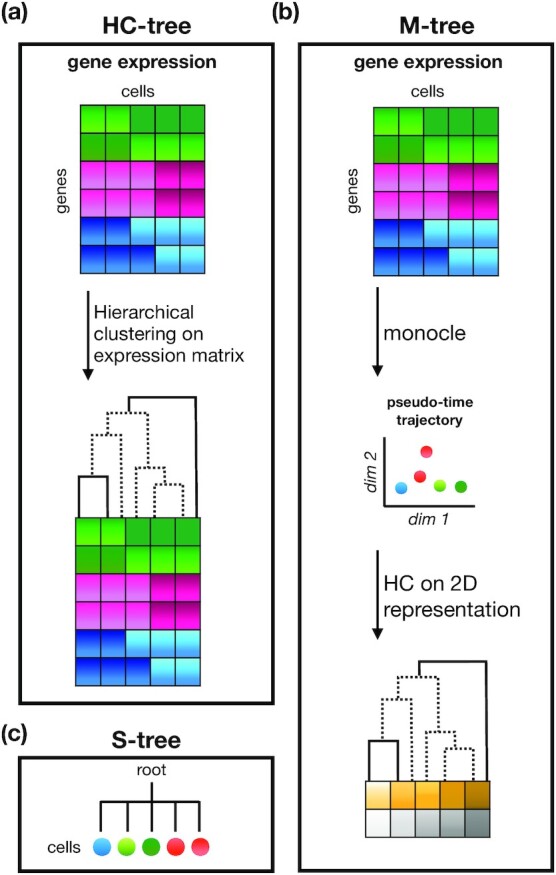
Schematic illustrations for the tree structures. (a) Performing hierarchical clustering on the gene expression matrix produces the HC-tree. (b) A pseudo-time trajectory is obtained using the Monocle software from the gene expression matrix. The resulting trajectory shown in a reduced 2D space is used to generate the M-tree structure. (c) Cells are connected to a root node forming a star-shaped tree, regarded as a baseline for the tree required models. dim: dimension.

The gene expression matrix is used to infer the tree structure of the tree-guided MTL models. For a sanity check of the models, we created a randomized gene expression matrix, to contrast the models trained on the real data with the random data. We generated several trees derived from the gene expression data to guide the optimization of the tree-guided MTL models. Fig. 2 summarizes the description of the tree structures listed below.

- **HC-tree**: We used the BuildTreeHC function of the LinearMTL package using the complete linkage and 1 − Pearsoncorrelation as the dissimilarity measure to apply hierarchical clustering on the real single-cell expression data. The clustering tree is then used to guide the tree-guided MTL models.
- **M-tree**: Coordinates of the single cells in the reduced dimension space (a matrix of size 2 × number of cells) derived from the Monocle model trained on the real data. Using the BuildTreeHC function, as described in HC-tree, we generated the tree structure from the coordinates in the reduced dimension space.
- **S-tree**: We constructed another tree that serves as a baseline for our tree-guided MTL models. The tree structure forms a star shape, with a root and as many child nodes as the number of cells. More precisely, let *k* be the number of cells. Then, the star tree has *k* + 1 nodes, labelled by 0, 1, ⋅⋅⋅, *k*, where 0 represents the root and the remaining nodes represent the *k* cells. Every non-root node has 1 and only 1 edge connecting it to the root. Clearly, the root has immediate links to other nodes, i.e., degree of *k*. This tree structure is considered baseline because it does not suggest any particular grouping of the cells, as they all are uniformly connected to the root.
- **R-tree**: To generate appropriate random data, we shuffled the gene expression profile of each single cell. Given that the genes are arranged in rows and cells in columns, for each column, we shuffled the gene expression values across the genes and then used this shuffled gene expression matrix as the input to the Monocle tool. Finally, we trained the tree-guided MTL model using the M-tree structure described above. Replacing the gene expression matrix in Fig. 2b with the shuffled expression matrix produces the R-tree set-up.

The implementation of our workflow can be accessed via the GitHub repository [26].

### Selection for heat map visualization

Because for the static features, several hundreds of TFs were included in the set and visualizing this many TFs makes the interpretation difficult, we decided to shrink this set by selecting those that pass a certain criterion. Essentially, for a given TF arranged in the rows of the coefficient matrix, we compute the sum of absolute regression coefficients for that TF across all cells. If this value is higher than our predefined threshold of 0.5, we keep that TF, or discard it otherwise, for visualization of TRIANGULATE result heat maps.

### Correlation analysis between expression and inferred TF activity

We define the TF-expr-cor as the Spearman correlation between the cell-specific expression of a TF and the inferred TF activity per cell (coefficients) of the TRIANGULATE model. We used a permutation approach to obtain a significance estimate for the correlation values.

First, we used the R-tree model, which is based on permuted expression values, to obtain an estimate of the regression matrix $\tilde{B}$. Second, we compute the Spearman correlation values between the permuted expression values and the computed regression coefficients in $\tilde{B}$ for each TF over all cells. This defines the null distribution of the TF-expr-cor values. In other words the TF-expr-cor values using the R-tree model define the null model.

This we can compare to an actual model. For example, using the HC-tree model, we computed the TF-expr-cor values. For our analysis (e.g., Fig. 8) we removed those values that lie within the range of TF-expr-cor values under the null model (Supplementary Fig. S1).

To illustrate the interesting TFs only, we further reduced the TF set by keeping those where the sign of correlation agreed with the sign of sum of coefficients across cells for a given TF.

(6) \begin{equation*} \mathrm{TF}_i: \left\lbrace \begin{array}{@{}l@{\quad }l@{}}\text{kept} & \text{if}\ \mathrm{Expr}(TF_i)\times \Sigma _{j}{B^j_i} > 0\\ \text{discarded}& \text{else}\, . \end{array}\right. \forall i \in \lbrace 1,\cdots ,n\rbrace , \end{equation*}

where the function Expr(TF_*i*_) denotes the log_2_-transformed TPM values of TF_*i*_ measured in single cells, $B^j_i$ is the coefficient corresponding to TF_*i*_ in cell *j*, and *n* is the number of TFs.

### Running SCENIC

SCENIC (SCENIC, RRID:SCR_017247) [11] was performed using the Python implementation pySCENIC (v0.9.19) [27] based on human TF ranking data base version 9 (motifs-v9-nr.hgnc-m0.001-o0.0 [28]) and human motif to TF annotation downloaded from [29] (v9) as outlined in the package manual.

## Results

We study the cell-specific association of regulatory elements by coupling distinct TF feature data with measurements of gene expression in single cells. For this purpose, we designed 3 different feature set-ups (static, dynamic, and ChIP-seq) representing the TF binding scores in various genomic regions (see Materials and Methods and Fig. 1). To conduct a supervised regression framework, we exploited 3 single-cell gene expression data sets (HLC/PHH, HSMM, and T cell) as the response variable. We performed several filtering steps to remove the low-quality data as described in Materials and Methods. After discarding these low-quality values from the HLC/PHH data, 238 cells remained with 14,142, 4,827, and 14,188 genes for static, dynamic, and ChIP-seq features, respectively. Similarly, for HSMM, the reduced data set contained 18,402 genes and 297 cells for the static features.

**Figure 3:**
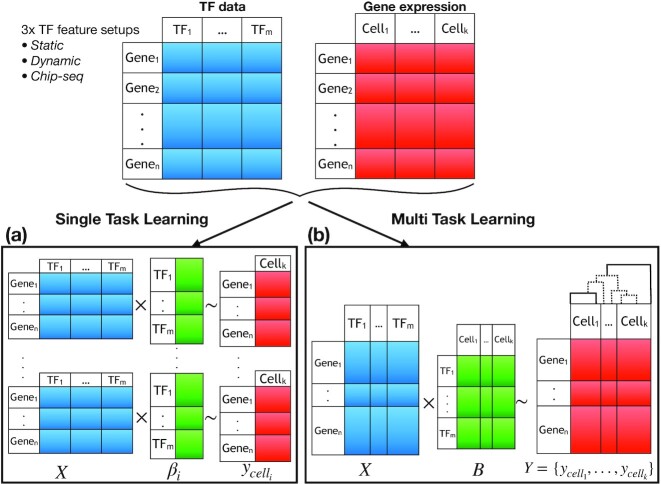
Schematic illustration of the learning set-ups, single- (a) and multi- (b) task learning. Common input files consisting of TF data (static, dynamic, or ChIP-seq) and single-cell gene expression are provided for both learning schemes. The rows of the feature matrix, *X*, are the genes for which one of the feature set-ups described previously would be used. The response matrix, *Y*, consists of the gene expression values measured in single cells. And finally, the coefficients matrix, *B*, establishes a linear association between the *X* and *Y*, where the rows indicate the features and columns the cells.

Given these data, we were able to train our models in 2 ways, as single tasks or combined with multi-tasking. As illustrated in Fig. 3a, in the STL case, each cell provides the response vector for an individual optimization problem solved through an elastic-net regularization (see Materials and Methods). Therefore, the total number of statistical models needed to be generated is equal to the number of cells in the given gene expression data set. On the other hand, when the MTL approach is used, the complete gene expression matrix is regarded as the response variable, where 1 model is created in the end. In this scenario, model coefficients are represented by a 2D matrix *B*, where each entry of *B* reflects the inferred activity of a particular TF in a specific cell.

### Tree-based models generally result in better performance

The tree-guided MTL models expect a tree structure to guide the model on how the tasks should be grouped when optimizing the objective function. However, the choice of the tree for the tree-guided MTL can be arbitrary because this tree is considered a hyperparameter set by the user. Therefore, we explored several trees for which we presumed they can represent the structure existing in the single-cell gene expression data.

As a trivial and straightforward choice, we applied hierarchical clustering directly on the gene expression data. The tree obtained from the hierarchical clustering was then used to train the tree-guided MTL models, to which we refer as “HC-tree." The next intuitive choice was to infer the tree structure from the pseudo-time ordering applied on the single cells because the differentiating cells should be placed closer to each other in this trajectory. Using the Monocle [20] tool (version 2), we were able to construct this trajectory for the single-cell expression data. Through traversing the trajectory obtained from Monocle, we built a tree representing the pseudo-time ordering of the cells. Because the transformation from the pseudo-time ordering to a tree can be arbitrary, we applied hierarchical clustering on the matrix holding the data for pseudo-time ordering and used the resulting tree for our tree-guided MTL models. We refer to this tree structure as “M-tree" (see Materials and Methods).

**Figure 4:**
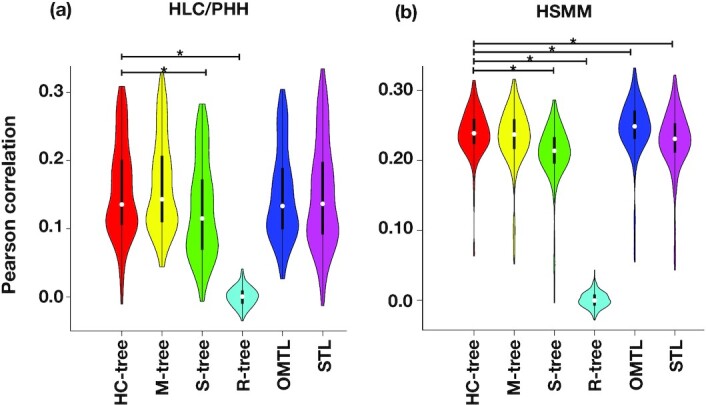
Comparison of MTL and STL models (shown on x-axis) on HLC/PHH (a) and HSMM (b) data sets. The comparison includes, hierarchical clustering (HC-tree), Monocle (M-tree), Star tree (S-tree), and random tree topology (R-tree) for tree-based multitasking methods, an ordinary multitasking approach without a tree regularization (OMTL), and an elastic net regularized single task learning per cell (STL). The y-axis is the Pearson correlation coefficient computed between the predicted and measured gene expression values on the test data. A 2-sided Mann-Whitney (unpaired) test with significance cut-off of 0.05 was performed between HC-tree and the other models. Pairs with significant differences are marked with an asterisk. See Supplementary Fig. S4 for a complete comparison of *P*-values on all pairs of models.

We further examined the performance of the tree-guided models with 2 other types of tree structures, “random" and “star" (see Materials and Methods). We used S-tree as a baseline for the tree-guided models because this structure imposes a uniform clustering of the cells (they all are at the same level relative to each other). Also, we introduced the R-tree to compare the performance of the models trained on the true data with the random data. Fig. 4 compares the performance of R-tree and S-tree with the HC-tree and M-tree models. These results suggest that the choice of hierarchical tree, performed on either the full gene expression data or the reduced space, is valid and reliable because they outperformed the R-tree and S-tree models.

Apart from the tree-guided models, we also generated the OMTL models to examine the efficiency of the tree-guided over OMTL models. Scatter plots provided in Supplementary Figs S2 and S3 allow us to compare the performance of the OMTL models with the tree-guided MTL models, where different trees are used.

In addition to the OMTL models, we trained individual single-task models, by providing the gene expression profile per cell as the response variable of each model (see Materials and Methods). The predictions obtained from each individual model were later used to compute the correlation values between the prediction and actual measurements of gene expression.

Fig 4 illustrates the distribution of Pearson correlation coefficients calculated between the predicted and measured values of gene expression for all the tree-guided MTL (HC-tree, S-tree, M-tree, and R-tree) as well as the OMTL and STL models, for both data sets.

The cell-wise comparison of all statistical models is provided as scatter plots in Supplementary Figs S2 and S3. These results indicate that in general, it is more difficult for the model to predict the gene expression of PHH cells, irrespective of the statistical model used. However, it is interesting to see that the STL models tend to be hindered more compared to the tree-guided MTL or the OMTL models, where they are essentially able to benefit from the information sharing among the tasks.

Given that the HC-tree and M-tree result in better-performing models in both HSMM and HLC/PHH data sets, we decided to pick one of these methods to proceed with the rest of our analysis. However, owing to the computational burden incurred by the additional step of running the Monocle software for inferring the trajectory in the M-tree case, we favored HC-tree over M-tree. Therefore, the following results are obtained from the HC-tree structure, which we hereinafter call TRIANGULATE.

### The impact of the number of cells on prediction results

Because the number of cells in the data set can play a crucial role in an MTL set-up, similar to what we proposed, we decided to challenge our TRIANGULATE by training it on fewer cells using the static features. Therefore, we downsampled the HLC/PHH cells using 5 different percentages of cells ($50\%$, $40\%$, $30\%$, $20\%$, and $10\%$). Table 1 shows the mean number of detected genes (the number of genes having expression of ≥1 in a given single cell) used for training the TRIANGULATE models. This table also provides the Pearson correlation measured between predicted and measured single-cell gene expression on test data of these downsampled HLC/PHH cells.

**Table 1:** Number of detected genes and Pearson correlation coefficients on test data for downsampled HLC/PHH cells based on 10–50% of the whole data

% of data	Mean ± SD	
	No. of detected genes	Correlation
10	4,522 ± 1,903	0.10 ± 0.04
20	4,545 ± 1,820	0.12 ± 0.05
30	5,025 ± 1,768	0.13 ± 0.06
40	4,960 ± 2,000	0.15 ± 0.07
50	4,818 ± 1,781	0.15 ± 0.07

Results obtained from the static features.

Overall, we observe that TRIANGULATE shows a robust performance when the number of cells is varied. However, there is an apparent trend of performance loss as the number of cells is decreased.

### The impact of feature types on the prediction results

We wanted to explore the associations of gene expression in single cells with features that are independent of the cell content or configuration. Therefore, we designed a feature set-up, which we named “static," to link the *cis*-regulatory characteristics of ∼700 TFs with the gene expression measurements of single cells (see Materials and Methods).

Fig. 1 schematically illustrates the genomic area where the static, dynamic, and ChIP-seq features are generated. In static features, for each transcription start site of a gene, the TF binding affinities are measured within the 2-kb window around the TSS. These affinity scores are used to form the feature matrix for the static set-up (Fig. 2a). Fig. 2b illustrates the dynamic set-up, where peaks are obtained from DNase1-seq data and used to identify the open chromatin regions in a 50-kb window around the TSS. The TF binding affinities are computed in the segments of this 50-kb window that correspond to the peaks. Finally, Fig. 2c shows the region where the reads of ChIP-seq data of 123 TFs are counted. The resulting measurements form the ChIP-seq features.

**Figure 5:**
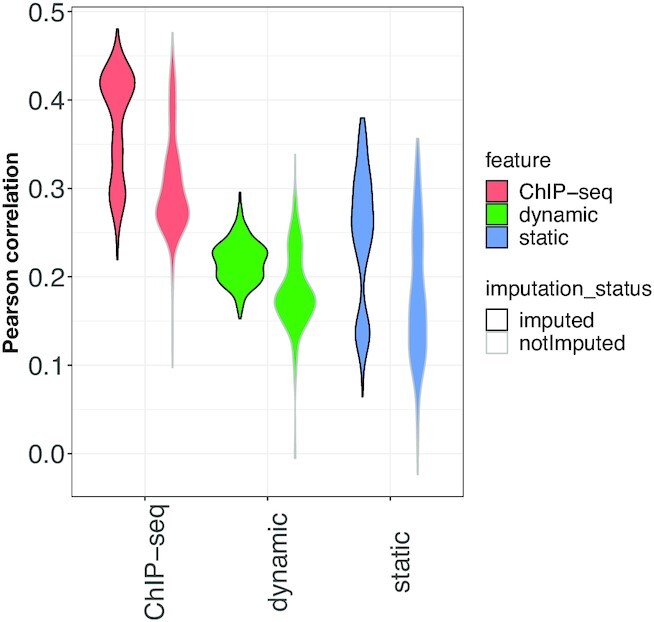
Comparison of different feature sets using the HC-tree MTL structure on the test partition of the HLC/PHH data (Pearson correlation, y-axis). ChIP-seq, dynamic, and static feature set-ups are indicated by red, green, and blue, respectively. The imputation status is indicated by different border colours, black for imputed (left) and gray for not imputed (right) for each feature set.

Fig. 5 describes the performance of the HC-tree MTL approach on the 3 feature set-ups for the HLC/PHH data set. We observed that TF features derived from measurements in HepG2 cells (dynamic or ChIP-seq features) showed better performance than the static feature set-up. Presumably, this reflects a more liver-specific association between a TF’s binding affinity and chromatin openness around a gene in those set-ups. Overall, the ChIP-seq features led to the most accurate models.

### Imputation generally improves the accuracy

Single-cell data are hindered by the inherent technical noise of so-called dropouts. Dropouts refer to genes that are falsely identified as zero-expressed. In simpler words, any zero that is observed in the expression count matrix of single-cell data can be viewed as either correctly or incorrectly identified as a silent gene due to the dropout effect. There have been several methods (e.g., [21,30,31]) that attempted to address this problem by imputing the missing expression values, but each of these methods has its own assumptions.

The results shown in previous sections are obtained using the original unaltered expression data. However, we were curious to find out how the results would change when we impute missing values potentially introduced by the dropout effect. Therefore, we imputed missing data using the scImpute tool ([21], see Materials and Methods) and repeated the experiments described with the difference of using the imputed expression values as the response matrix.

Fig. 5 provides an overview of the performance of the HC-tree MTL model on the 3 feature set-ups, comparing the use of original or imputed scRNA-seq counts. These results reveal that the imputation enhances the prediction accuracy, regardless of the feature set-up. It is interesting to observe that for the dynamic set-up, not only the correlation values are increased, but also the distribution of these values is changed in favor of having a smaller variance across the cells. The change of distribution is notable for the other 2 set-ups as well, but that does not necessarily lead to a smaller variance.

### Cell type–specific TF activities inferred from the model coefficients

**Figure 6:**
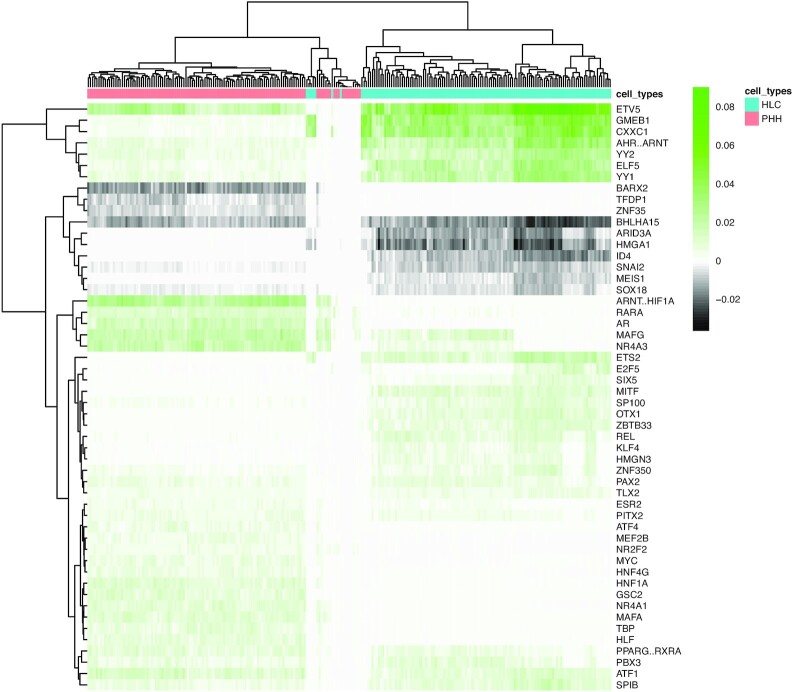
Coefficients of the tree-guided MTL model using the HC-tree structure. Heat map illustrating the top features (see Materials and Methods) derived from the tree-guided MTL trained on the static features to predict the unimputed gene expression in HLC/PHH cells.

Observing such difference in the prediction accuracy inspired us to inspect the model coefficients that correspond to the activity of TFs in cells. The heat map in Fig. 6 depicts the coefficients of the top features (see Materials and Methods) derived from the HC-tree model trained on the static features to predict the gene expression in HLC/PHH cells.

In this heat map, it can be noted that, first, the cells are clearly clustered according to the model coefficients, separating the HLC and PHH cell types. Second, these results show certain groups of TFs playing distinct roles in regulating gene expression in cell subpopulations. For instance, the TF YY2 holds positive coefficient values for the HLC cells, whereas its values for the PHH cells are negative. This is interesting because YY2 may perform a dual effect on gene expression; i.e., it can both repress and activate transcription [32].

On the other hand, HNF1A, which is essential for the expression of various liver-specific genes, was considered less relevant for the HLC cells by the model, as it has mostly assigned zero to coefficients corresponding to HNF1A. However, HNF1A holds positive coefficient values for the PHH cells. GMEB1 is another factor that shows a variable activity between the 2 cell types. Data from the Human Protein Atlas suggests it to be a prognostic marker in liver cancer [33].

In addition, we observe that PBX3 and MAFG are TFs that seem to be active for both cell types according to our model. It has been shown that PBX3 plays a crucial role in the transcriptional program of human liver tumour-initiating cells [34]. Liu and co-workers [35] observed that MAFG is positively correlated with the progression of tumour cells especially in patients with cholangiocarcinoma and hepatocellular carcinoma.

We additionally inspected the model coefficients to check whether the 2 cell types were still separable on the downsampled data sets introduced before (Table 1). Supplementary Fig. S5 shows that TRIANGULATE was able to assign the coefficients of the model appropriately according to the cell types in the downsampled data.

### Comparison of results with SCENIC

Because SCENIC [11] is also able to produce cell-specific TF activity scores, we were interested in comparing the results of our models with the AUC values that SCENIC computes to represent the TF activity (see Materials and Methods). Because the AUC values are positive, SCENIC can only infer positive associations, in contrast to our method, which is able to deduce negative associations as well. As a result, comparing the activity matrix directly (Supplementary Fig. S6) was not a meaningful approach.

**Figure 7:**
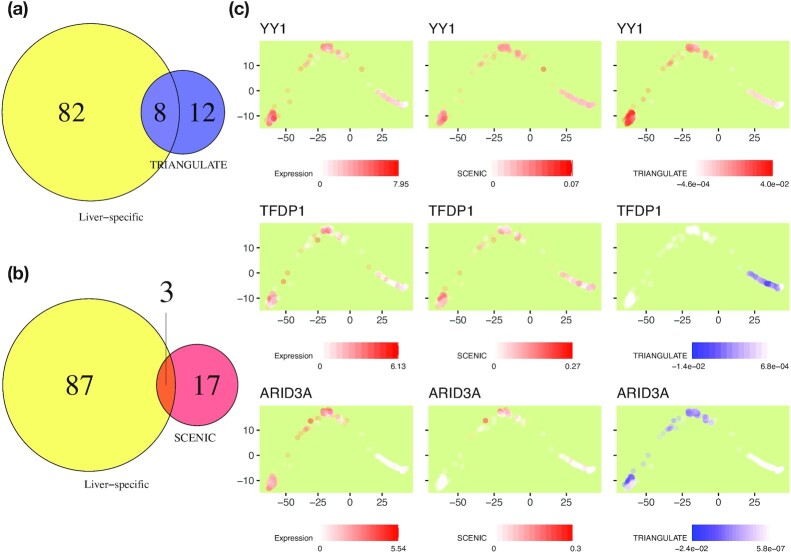
Comparison of inferred TF activities. Venn diagram depicting the number of overlapping TFs between liver-specific and each of (a) SCENIC and (b) TRIANGULATE models. The top 20 TFs that were identified to be active are used for the comparison of SCENIC and TRIANGULATE. (c) Exemplary TF trajectories are shown in the single-cell trajectory based on the expression of TF (left), the AUC score obtained from SCENIC (middle), or the coefficients of the TRIANGULATE model (right).

For this purpose, we defined per TF activities by adding up their activity scores (AUC values for SCENIC and scaled coefficient values for TRIANGULATE) across cells. The top 20 active TFs obtained from each approach were compared with a set of known liver-specific TFs previously collected through literature search [18]. The number of overlapping TFs between the liver-specific set and top 20 active TFs obtained from TRIANGULATE is shown in Fig. 7a (similarly for SCENIC in Fig. 7b). These results indicate that TRIANGULATE is able to identify more liver-specific TFs than SCENIC among the top 20 active TFs.

We further noticed that YY1, TBP, HNF4G, KLF, and CEBPA were the 5 liver-specific TFs that only TRIANGULATE was able to identify among its top 20 TFs while SCENIC could not.

We were also interested in identifying the TFs that showed a significant difference in their inferred activity between the HLC and PHH cell types. We applied a (2-sided) Mann-Whitney test using the significance cut-off of 0.1 on the multiple-testing–corrected *P*-values (Benjamini-Hochberg method) to select the TFs that are significantly different between the HLC and PHH cells. In our proposed method, this test was applied on the model coefficients and in SCENIC on the AUC values obtained for the HLC/PHH cells. The Venn diagram shown in Supplementary Fig. S7 illustrates the number of TFs common between the liver-specific TF set, SCENIC, and the TRIANGULATE approach. It can be seen that TRIANGULATE and SCENIC have 26 and 28 TFs in common with known liver-specific TFs, respectively. This indicates that our approach is able to find liver-specific TFs and mostly agrees with the results of SCENIC when considering differences in regulation between the 2 cell types.

The agreement we observed between the liver-specific TFs and top active TFs suggested by SCENIC and TRIANGULATE inspired us to advance our investigation by analysing the inferred TF activities in each individual cell.

Using the pseudo-time ordering of the cells obtained from Monocle, we displayed the cells in the 2D trajectory space and marked each cell on the basis of their inferred activity obtained from SCENIC or TRIANGULATE, as well as the expression of the TF in single cells (Supplementary Fig. S8 and Fig. 7c). It should be noted that the scores that SCENIC obtains are a function of a TF’s gene expression, which is used in the first step of the method. Thus, it is not surprising to see that the SCENIC AUC scores often agree well with the trend observed in TF expression values, but rather confirms that the approach works as intended. As shown in Fig. 7c, YY1 appears to be expressed across the single-cell trajectory. This behaviour is reflected in the inferred TF activities by SCENIC and TRIANGULATE. However, as mentioned earlier, this TF did not appear among the top 20 active TFs for SCENIC. This is due to the very small AUC values that SCENIC computed for YY1 (maximum of 0.07).

**Figure 8:**
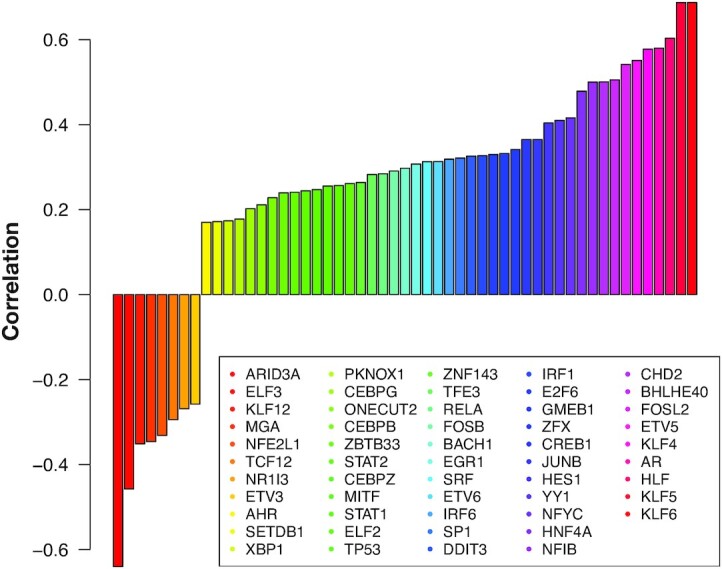
Spearman correlation between a TF’s gene expression and inferred TF activity from TRIANGULATE over all cells in the HLC/PHH data set. TFs are sorted by correlation, and correlation values smaller than obtained by a permutation analysis are not shown (see Methods).

Another interesting example is TFDP1. We observed that TRIANGULATE assigned negative coefficients to the PHH cells of TFDP1 (Figs 6 and 7c). It has been shown that TFDP1, together with E2F1, is involved in regulating hepatocellular carcinoma cells through a knockdown experiment that confirmed a physical interaction of KPNA2 with E2F1 and TFDP1 [36].

We additionally found that ARID3A is among the TFs that had negative activity consistently across the single cells. We found evidence in the literature that ARID3A has been identified as a repressor in embryonic regulation [37,38].

From the trajectory plots provided in Fig. 7c, it can be seen that the activities of TFDP1 and ARID3A are concentrated on different subsets of cells. As previously mentioned SCENIC cannot infer negative TF activity, which can explain the small AUC scores (maximum of 0.27) assigned to the cells for these 2 TFs.

It is worth mentioning that the coefficient values we obtained from TRIANGULATE are in general very small. This is due to the large number of features each data point has. Because our model exploits ∼700 TFs in its feature space, among which many are non-zero (selected by the model), the values assigned to these coefficients need to be small so that the linear combination of the coefficients and feature data falls within the range of response values. Because we want to explore the negative associations, we show the original coefficients in Fig. 7.

To further evaluate and compare the performance of TRIANGULATE with that of SCENIC, we acquired another data set of single-cell gene expression (Supplementary Methods). These data consist of the CD4^+^ T cells from a patient with liver cancer (accession No. GSM2602298) [39]. A total of 176 cells are annotated as normal and 230 cells are annotated as tumour. We ran SCENIC and TRIANGULATE in the aforementioned manner to identify the top 20 active TFs. We also prepared a list of T cell–specific TFs from the literature provided in Supplementary Table S1. By overlapping the set of known TFs regulating T cells with the top 20 active TFs obtained from TRIANGULATE and SCENIC, we were able to find 15 and 8 TFs, respectively. The Venn diagrams shown in Supplementary Fig. S9 illustrate this overlap for both approaches. Similar to the analysis of the HLC/PHH data set, TRIANGULATE was able to find more cell type–specific TFs than SCENIC.

### TF expression and its inferred activity

Because we often observed agreement between the expression and TRIANGULATE coefficients (scores), we decided to systematically assess this similarity. To inspect how much the inferred TF activities agree with the expression of a TF’s gene in single cells, we designed a correlation analysis to evaluate the similarity between these 2 quantities. As described in Materials and Methods, we introduce TF-expr-cor, the Spearman correlation between the coefficients obtained from the TRIANGULATE model and the log_2_-transformed TPM values representing the expression of TFs. Fig. 8 illustrates the TF-expr-cor values for a subset of TFs that had higher values than a null model (the R-tree model). According to this analysis, ARID3A and KLF6 have the smallest negative and largest positive TF-expr-cor values, respectively.

Contrasting the gene expression signal of ARID3A shown in Fig. 7c with the inferred activity from TRIANGULATE delineates the strong negative correlation between the gene expression of ARID3A and its TRIANGULATE coefficients that we observed in Fig. 8.

For the TFs whose correlation values were relatively high, we also observed a less sparse signal (fewer zeroes) in their corresponding gene expression data. This observation holds for both positive and negative correlations. However, one should not expect high correlations for all TFs with high inferred activities. Still, if both quantities correlate, this may point to regulators of interest concerning the studied data set.

## Discussion

The discrepancies observed among the gene expression profiles in single cells, trivially, hint at the existence of specific differences in the transcriptional regulatory program. Devising computational methods that are able to infer associations between gene expression in single cells and *cis*-regulatory motifs, as well as epigenetic characteristics, has attracted the attention of researchers in the field (e.g., [8–11]).

In this work we analysed a regression formulation, where TF features based on sequence motif matches, bulk ChIP-seq peaks, or DNase1-seq peaks were used to predict gene expression in individual cells. Previously, such regression formulations were only performed in the context of bulk gene expression prediction. Obviously, for single-cell data this is a harder problem because most of the single-cell data available are very sparse with only a few read counts per gene, if any. These technical limitations are challenging to address.

On the 2 data sets we investigated, we found that the correlation on test data is not overwhelming, but it is hard to come up with an expected correlation. Using similar features in a regression of bulk RNA-seq, coupled with epigenomic data sets, led to correlation coefficient values of ∼0.3–0.6 [18], but with Dnase1-seq and RNA-seq data obtained from the same cells.

We wondered whether imputation would improve the correlation on the test set. For that, we generated our statistical models on the imputed gene expression data. The results displayed in Fig. 5 consistently show an improvement in accuracy for the imputed models. We think that by imputing the missing values of gene expression, a stronger connection may have been established between the TF and gene expression data. Therefore, the signal existing in the features (TF data) better reflects the variances in the response (gene expression).

We designed a general framework for establishing cell-specific associations through using tree-guided MTL [24]. This framework benefits from both the information sharing delivered by multi-tasking and also grouping the cells according to the tree structure provided as an additional input. The choice of the tree is a hyperparameter (user-dependent). We tried exploring different tree structures, and our findings indicate that the performance of the models is influenced by this choice. In addition, we see that depending on the data set, the best-performing model is generated from different tree structures. Therefore, it is difficult to suggest an all-purpose tree inference approach that would work best for all data sets. Besides, we think that the optimization model could be modified such that it is not restricted to a tree structure but an arbitrary graph for grouping the tasks (cells).

Another area for improvement is the distribution assumption. The Gaussian distribution used in the current optimization does not best address the specifics of count data, nor the single-cell measurement noise such as dropouts. As a future work, we propose incorporating the negative binomial distribution to better account for these issues.

To check whether the accuracy of our models would improve when a non-linear learning set-up is applied, we designed a vanilla artificial neural network (Supplementary Methods). By comparing the median of the correlation between our linear models and this neural network, as shown in Supplementary Fig. S10, we did not observe any significant improvement achieved by the neural network models. Thus, another direction would be to adapt more sophisticated neural network architectures, such as [5], to single-cell data.

An obvious advantage of the linear modelling approaches is the straightforward interpretation of TF regression coefficients. By inspecting the coefficients of our models, we were able to pinpoint distinct TFs that show cell type–specific regulation in HLC/PHH cells and showed that many liver-specific regulators could be inferred in this way.

## Conclusion

The problem of identifying cell-specific regulatory elements is a difficult task, mainly owing to the technical noise in the single-cell data. However, in this study, we built several statistical models that lead to stable feature selection, which provides interpretable results. Also, our method can be used directly to incorporate various approaches for designing TF features, such as TF binding affinities and ChIP-seq signals.

As a future work, it would be interesting to extend this study using paired scRNA and single-cell open-chromatin data [40–42], particularly for the design of dynamic features. Using this type of data allows us to estimate the TF activity in accessible chromatin regions defined on the basis of individual single cells.

## Availability of Source Code and Requirements

- Project name: TRIANGULATE
- Project home page:  https://github.com/SchulzLab/Triangulate
- Operating system: x86_64-pc-linux-gnu (64-bit)
- Programming language: R (version 3.4.4)
- Other requirements: monocle (2.9.0), stringr (1.4.0), LinearMTL (0.2.0), doParallel (1.0.15)
- License: MIT

## Availability of Supporting Data and Materials

Preprocessed feature and expression matrices are available in the *GigaScience* GigaDB database [43].

## Additional Files

Supplementary Methods.

Supplementary Figure S1. Correlation between TF’s gene expression and inferred TF activity (R-tree).

Supplementary Figure S2. Correlation between predicted and measured gene expression per cell (HLC/PHH).

Supplementary Figure S3. Correlation between predicted and measured gene expression per cell (HSMM).

Supplementary Figure S4. Comparison of statistical models on test data of static features.

Supplementary Figure S5. TRIANGULATE model coefficients on down-sampled cells.

Supplementary Figure S6. SCENIC AUC values obtained on the HLC/PHH data

Supplementary Figure S7. Number of overlapping TFs significantly distinct between the PHH and HLC cell types

Supplementary Figure S8. 2D trajectories obtained from Monocle and colored based on the inferred TF activity

Supplementary Figure S9. Overlap between the set of T cell-specifi TFs and the top 20 active TFs

Supplementary Figure S10. Median of the test correlation values for different types of statistical models.

Supplementary Table S1. List of known TFs are involved in regulating T cells

## Abbreviations

AUC: area under the curve; bp: base pairs; ChIP-seq: chromatin immunoprecipitation followed by sequencing; DNase-seq: DNase I hypersensitive sites sequencing; EGA: European Genome-phenome Archive; HLC: hepatocyte-like cells; HSMM: human skeletal muscle myoblast; kb: kilobase pairs; MTL: multi-task learning; OMTL: ordinary multi-task learning; PHH: primary human hepatocyte; SCENIC: Single-Cell rEgulatory Network Inference and Clustering; STL: single-task learning; TF: transcription factor; TPM: transcripts per million; TRAP: transcription factor affinity prediction; TRIANGULATE: tree-guided estimation of single-cell regulation; TSS: transcription start site.

## Competing Interests

The authors declare that they have no competing interests.

## Funding

This work has been supported by the DZHK (German Centre for Cardiovascular Research, 81Z0200101) and the DFG Clusters of Excellence on Multimodal Computing and Interaction [EXC248] and Cardio-Pulmonary Institute (CPI) [EXC 2026], and the DFG SFB/TRR 267 Noncoding RNAs in the Cardiovascular System.

## Authors' Contributions

F.B.A. and M.S. conceived and designed the project. F.B.A. implemented the TRIANGULATE workflow and produced all figures/plots; F.S. generated the TF feature data; K.K. ran SCENIC on the HLC/PHH and T-cell data sets; F.B.A. analysed the SCENIC results and produced the TF-expr-cor analysis; T.H. implemented the linearMTL package; D.F., G.G., K.L., P.N., J.H., and J.W. were involved in generation and/or primary processing of the HLC/PHH single-cell gene expression data. F.B.A. and M.S. wrote the draft manuscript. All authors approved the final manuscript.

## Supplementary Material

giaa113_GIGA-D-20-00071_Original_Submission

giaa113_GIGA-D-20-00071_Revision_1

giaa113_Response_to_Reviewer_Comments_Original_Submission

giaa113_Reviewer_1_Report_Original_Submission Wei Chen -- 4/28/2020 Reviewed

giaa113_Reviewer_1_Report_Revision_1 Wei Chen -- 8/27/2020 Reviewed

giaa113_Reviewer_2_Report_Original_Submission Longqi Liu -- 5/23/2020 Reviewed

giaa113_Supplemental_File
