# Supplementary material for: Prediction of single-cell gene expression for transcription factor analysis
Source: Gigascience. 2020 Oct 30;9(11):giaa113. doi: 10.1093/gigascience/giaa113 (PMC7596801; doi:10.1093/gigascience/giaa113)
Supplement: giaa113_GIGA-D-20-00071_Revision_1 [file giaa113_giga-d-20-00071_revision_1.pdf]

GigaScience

Prediction of single cell gene expression for transcription factor analysis

--Manuscript Draft--

**Manuscript Number:**	GIGA-D-20-00071R1
**Full Title:**	Prediction of single cell gene expression for transcription factor analysis
**Article Type:**	Research
**Funding Information:**	Deutsches Zentrum für Herz-Kreislaufforschung (81Z0200101) — Not applicable<br>Deutsche Forschungsgemeinschaft (EXC248, EXC 2026, SFB/TRR 267) — Not applicable
**Abstract:**	Single cell (sc) RNA-sequencing is a powerful technology to discover new cell types and study biological processes in complex biological samples. A current challenge is to predict transcription factor (TF) regulation from scRNA data.<br><br>Here, we propose a novel approach for predicting gene expression at the single cell level using cis-regulatory motifs as well as epigenetic features. We designed a tree-guided multi-task learning framework that considers each cell as a task. Through this framework we were able to explain the single cell gene expression values using either TF binding affinities or TF ChIP-seq data measured at specific genomic regions. TFs identified using these models could be validated by literature.<br><br>Our proposed method allows us to identify distinct TFs that show cell-type specific regulation. This approach is not limited to TFs, but can use any type of data that can potentially be used in explaining gene expression at the single cell level to study factors that drive differentiation or show abnormal regulation in disease. The implementation of our workflow can be accessed under an MIT license via: https://github.com/SchulzLab/Triangulate.
**Corresponding Author:**	Fatemeh Behjati Ardakani<br>Goethe-Universitat Frankfurt am Main<br>Frankfurt am Main, GERMANY
**Corresponding Author Secondary Information:**	
**Corresponding Author's Institution:**	Goethe-Universitat Frankfurt am Main
**Corresponding Author's Secondary Institution:**	
**First Author:**	Fatemeh Behjati Ardakani
**First Author Secondary Information:**	
**Order of Authors:**	Fatemeh Behjati Ardakani
	Kathrin Kattler
	Tobias Heinen
	Florian Schmidt
	David Feuerborn
	Gilles Gasparoni
	Konstantin Lepikhov
	Patrick Nell
	Jan Hengstler
	Joern Walter
	Marcel H Schulz

Powered by Editorial Manager® and ProduXion Manager® from Aries Systems Corporation

Order of Authors Secondary Information:	
Response to Reviewers:	Reviewer reports:<br>Reviewer #1: In the manuscript, Ardakani et.al. described a novel computational framework that can predict gene expression level at single cell level based on scRNA-seq data as well as the inferred TF activity. They demonstrated its superior performance by applying it in two datasets. Overall, the manuscript is well written except a couple of places with minor grammar errors. I believe the tool developed by them will be of interest to the single-cell genomics community. I have only two minor comments/suggestions.<br>##<br>1. In fig4a. it is unclear whether the Tree-guided outperforms OMTL and STL. How would OMTL compare to STL? In Fig4b, visually the difference between HC-tree band OMTL is not that big, how would M-tree compare to OMTL? How would S-tree compare to OMTL and STL?<br><br>We apologize for not providing the statistics on all pairs, in the first version of the manuscript. We now added figures to our supplemental materials that provide the Mann-Whitney test p-values on all pairs of models (Supp. Fig. 3).<br><br>##<br>2. The author should discuss how the performance of their tool would be potentially affected by the features of scRNA-seq datasets, e.g. cell numbers, number of cells in different clusters, No. UMI/cell,No. genes/cell and etc.<br><br>This is a good idea, thank you. We added a new subsection in the Results section of the manuscript (The impact of the number of cells on prediction results) on the use of TRIANGULATE on single cell datasets of different scale and quality.<br><br><br>Reviewer #2: In this manuscript the authors developed a workflow to identify transcription factors (TFs) that may have cell type-specific regulation from single-cell RNA-seq (scRNA-seq) data. To achieve this, they tested many different methods: to generate proper TF feature matrices they use static, dynamic and ChIP-seq features; for statistical learning frameworks they use single- and multi-task learning methods. Furthermore, they generated several trees to guide the optimization of the tree-guided MTL models. By systematic comparisons, they found HC-tree and ChIP-seq showed better performing results in the data sets they tested. And finally, they compared the results with SCENIC, which is one of the most commonly used software to explore gene regulatory networks within scRNA-seq data. They found that TRIANGULATE had better performance in TF motif prediction and more important, TRIANGULATE can predict negatively correlated TF-gene pairs, which is a unique feature to TRIANGULATE. This manuscript is suitable for GigaScience, however, some issues need to be addressed before it can be considered for publication.<br>##<br>1.The authors tested only two cell types (HLC and PHH), can TRIANGULATE be applied to other data with similar performance as well? In other words, in addition to HLC and PHH, how is the performance of TRIANGULATE compared to SCENIC?<br><br>Thanks for raising this point. To address that, we gathered T cells from a human HCC patient (GSM2602298) with normal and tumor cell annotations. We ran TRIANGULATE and SCENIC on this data, and the results are described in the Results section (Comparison of results with SCENIC subsection) of the manuscript.<br>In brief, our method performs similar to the HLC/PHH dataset in terms of prediction accuracy measured in Pearson correlation. To compare it with SCENIC and a set of known T cell specific TFs, we overlapped the set of known T cell specific TFs with the set of top 20 active TFs from TRIANGULATE and SCENIC, where we found 15 and 8 hits, respectively.<br><br>##<br>2.The title seems too broad and it is hard to get the main point of this manuscript.<br><br>We are sorry that the manuscript's title did not seem very right to the point, but we decided to choose the "Prediction of single cell gene expression for transcription factor analysis" title for the following reasons:

	- A predictive model is proposed that predicts the single cell gene expression, therefore “Prediction of single cell gene expression”<br>- This model enables us to infer and analyze the transcription factor activity within these single cells, therefore “for transcription factor analysis”.<br>Thus, we think that the title reflects the core contribution of our manuscript.<br>##<br>3.The authors used 2 kb to define the promoter of a certain gene. How is this threshold selected? This is important because it affects the performance of TRIANGULATE.<br><br>The threshold is selected on the basis of previous work with sequence based motif prediction in the context of gene regulation, where we found that including motif hits in a window larger than 2kb is not working well.<br>##<br>4.It would be better to import the matrix directly instead of using Monocle, because the users may have their preferred methods to filter their data.<br><br>Yes, that is a valid point. We added the option to directly import the matrix to Triangulate. The details are added to the GitHub readme page.<br>##<br>5.The interface and description of TRIANGULATE in GitHub are simple. The authors should provide a more detailed tutorial and add more annotations, such as the meaning of the functions and variables listed on the website.<br><br>We are sorry that the description was insufficient. We improved the description of the scripts, their arguments, and variable names, as well as the purpose of those scripts as part of the snakemake workflow rules.
**Additional Information:**	
**Question**	**Response**
Are you submitting this manuscript to a special series or article collection?	No
**Experimental design and statistics**<br><br>Full details of the experimental design and statistical methods used should be given in the Methods section, as detailed in our Minimum Standards Reporting Checklist. Information essential to interpreting the data presented should be made available in the figure legends.<br><br>Have you included all the information requested in your manuscript?	Yes
**Resources**<br><br>A description of all resources used, including antibodies, cell lines, animals and software tools, with enough information to allow them to be uniquely identified, should be included in the Methods section. Authors are strongly encouraged to cite Research Resource	Yes

Powered by Editorial Manager® and ProduXion Manager® from Aries Systems Corporation

Identifiers (RRIDs) for antibodies, model organisms and tools, where possible.<br><br>Have you included the information requested as detailed in our Minimum Standards Reporting Checklist?	
**Availability of data and materials**<br><br>All datasets and code on which the conclusions of the paper rely must be either included in your submission or deposited in publicly available repositories (where available and ethically appropriate), referencing such data using a unique identifier in the references and in the "Availability of Data and Materials" section of your manuscript.<br><br>Have you have met the above requirement as detailed in our Minimum Standards Reporting Checklist?	Yes

Manuscript
Click here to access/download;Manuscript;Triangulate_main.pdf

OXFORD (GIGA)[n] SCIENCE

GigaScience, 2020, 1–12

doi: xx.xxxx/xxxx
Manuscript in Preparation
Paper

PAPER

Prediction of single cell gene expression for transcription factor analysis

Fatemeh Behjati Ardakani[1,2,3,4*], Kathrin Kattler[5], Tobias Heinen[2,3], Florian Schmidt[1,2,3,4], David Feuerborn[6], Gilles Gasparoni[5], Konstantin Lepikhov[5], Patrick Nell[6], Jan Hengstler[6], Jörn Walter[5] and Marcel H. Schulz[1,2,3*]

[1]Institute for Cardiovascular Regeneration, Goethe University, 60590 Frankfurt am Main, Germany and [2]Cluster of Excellence MMCI, Saarland University, Saarland Informatics Campus, 66123 Saarbrücken, Germany and [3]Max Planck Institute for Informatics, Saarland Informatics Campus, 66123 Saarbrücken, Germany and [4]Graduate School of Computer Science, Saarland University, Saarbrücken, Germany and [5]Department of Genetics, Saarland University, 66123 Saarbrücken, Germany and [6]Leibniz Research Centre for Working Environment and Human Factors (IfADo), 44139 Dortmund, Germany

*fbehjati@med.uni-frankfurt.de; marcel.schulz@em.uni-frankfurt.de

Abstract

Single cell (sc) RNA-sequencing is a powerful technology to discover new cell types and study biological processes in complex biological samples. A current challenge is to predict transcription factor (TF) regulation from scRNA data. Here, we propose a novel approach for predicting gene expression at the single cell level using cis-regulatory motifs as well as epigenetic features. We designed a tree-guided multi-task learning framework that considers each cell as a task. Through this framework we were able to explain the single cell gene expression values using either TF binding affinities or TF ChIP-seq data measured at specific genomic regions. TFs identified using these models could be validated by literature. Our proposed method allows us to identify distinct TFs that show cell-type specific regulation. This approach is not limited to TFs, but can use any type of data that can potentially be used in explaining gene expression at the single cell level to study factors that drive differentiation or show abnormal regulation in disease. The implementation of our workflow can be accessed under an MIT license via: https://github.com/SchulzLab/Triangulate.

Key words: single cell RNA-seq; Gene regulation; Multi-task Learning; Regulatory networks

Background

Single cell sequencing has become a powerful tool to study gene expression patterns in different cellular contexts, such as cell differentiation, complex tissues, and disease. It is an open question how to best use single cell RNA (scRNA-seq) data to infer cell-specific transcriptional regulatory programs.

Many methods have been developed that use gene expression data of pooled cell samples (bulk) to infer cell-specific transcription factor (TF) regulation. These methods often use the idea to decompose or associate variance in measured gene expression data with putative TF target gene sets to infer TF activity. To name a few examples, such approaches include network component analysis [1], methods for predicting gene expression values from TF motifs [2, 3], combined with epigenetic [4] or chromatin conformation data [5, 6].

As scRNA-seq data protocols are becoming more widely adopted, novel methods have been developed that learn TF reg-

Compiled on: August 20, 2020.
Draft manuscript prepared by the author.

Key Points

- Establish a novel framework for using transcription factor motif, ChIP-seq or epigenetics data to predict expression in single cells
- Single cell-specific transcription factor activities can be inferred for positive and negative associations
- Application to hepatocyte single cell data reveals known and novel regulators

ulation by making use of the large number of cells obtained in current experiments, an overview was done by [7]. For example the ACTION method [8] is an approach that identifies marker genes for each cell cluster from scRNA-seq data. It then uses a TF enrichment approach, using known TF-gene interactions, to determine TFs of regulatory importance for a set of marker genes in each cell cluster identified. Another approach suggested by [9], uses a Kalman filter to model expression changes of single cell clusters in differentiation processes by explicitly modelling the contribution of TFs in cell state transitions. scRNA-seq data was also used to build neuronal network classifiers that predict TF-gene target relationships by utilizing other types of information such as ChIP-seq data [10].

SCENIC [11] is a widely used method for scRNA-seq data analysis. It uses a three-step approach to infer regulatory networks. First, TF associations are inferred using regression trees that learn single cell gene expression from expression of TF-encoding genes. Second, these co-expression modules are tested for TF enrichment, such that significantly enriched TFs are used to define a TF regulon, by restricting to direct targets using motif information (window of 10 kb around the TSS or 500 bp upstream the TSS). Third, the positively associated regulons are then used to be incorporated with the single cell data. Through this step, the activity of each regulon in each cell is evaluated by calculating an AUC score, integrating the expression ranks across all genes in a regulon. Finally, these scores are used to create the desired activity matrix as output of their workflow. The authors mention that their approach could not find significant TF regulons where genes are negatively associated with TF expression, and thus the ranking in step three is limited to find TF regulons among the highest expressed genes in a cell.

Later, [12] exploited SCENIC and modified it by defining a Jensen-Shannon divergence based score to assess the cell type specificity of the regulons. By considering the regulons having high values of such customized score, they were able to infer both known and novel regulatory elements in the mapped mouse cell atlas.

One of the appealing aspects of the approach by SCENIC is that it is able to infer a TF activity per cell. However, as scRNA-seq data is noisy, this inference is challenging and as stated above, negative associations are not possible on a single-cell level in this way.

A widely adopted approach to overcome noise in challenging machine learning applications is the use of multi-tasking. In the context of bulk RNA-seq analysis several regression approaches that associate regulatory features with gene expression in a multi-tasking framework have been proposed [13, 14, 15, 16].

In this work, we introduce *TRIANGULATE*, a tree-guided multi-tasking approach for inferring gene regulation in single cells. This work is conceptually similar to SCENIC [11] as it derives a TF activity score per cell, but it is methodologically different. Similar to SCENIC, we study the associations between single cell gene expression and transcription factors. We train statistical models, where the expression measurements of genes across single cells are considered as the tasks in a multi-task-learning (MTL) setup. In contrast to SCENIC, we compute the binding affinities of many TFs instead of relying on the TF's gene expression and explore the use of alternative ways for measuring TF activity, for example using bulk epigenetic data or TF ChIP-seq data of related cells.

We trained our models on three single cell gene expression data sets, a data set comprising primary human hepatocytes (PHH) and *in vitro* differentiated Hepatocyte-Like cells (HLC), a data set of human skeletal muscle myoblasts (HSMM), and the third a data set of normal and tumor samples from T cells of a liver cancer patient. We inspected the coefficients of these models to identify interesting sets of features that best explain the gene expression in single cells. In addition, we compared the MTL results with standard univariate response regression models. These results indicate that the MTL models that integrate the information among all single cell gene expressions not only produce more interpretable models, but also often lead to higher accuracy.

Materials and methods

Generating TF feature matrices

In this section, we explain how the feature and response matrices were generated for our statistical models. We define $F_S \in \mathbb{R}^{n\times p}$ to be the feature matrix representing the TF data measured for n genes, arranged at the rows, and p TFs, arranged at the columns. We generate the TF data in three different ways, as described below. In addition, we use the single cell RNA-seq data as the response variable for our statistical models. After applying the filtering steps described below, we apply a log2-transform to all feature and response matrices prior to the model fitting phase.

Static features

TRAP [17] was run to quantify the binding affinities of 726 TFs at the promoter area defined by a window of size 2 kb centered at the transcription start site (TSS) using Position Weight Matrices from the TEPIC repository [18, 19]. These affinity values form the *static* features.

Dynamic features

Using TEPIC version 2.0 [19], the binding affinities of 726 TFs were measured in peaks defined based on the DNase1-seq data within the 50 kb window around the TSSs of HepG2 cells produced by DEEP [18], and mapped against human genome hg38. The contribution of TF motifs in DNase1-seq peaks are weighted using an exponential decay function in the 50kb window as previously introduced [18]. In contrast to the *static* case, in this setup, we additionally include three extra features representing the number of DNase peaks (*Peak_Counts*), the length of the open region (*Peak_Length*), and the aggregated DNase1-seq signal (*Peak_Signal*) computed within the 50 kb window around the TSS. A previous study showed that including these three features improves feature selection for gene expression prediction [4]. Because this particular type of feature is derived

from the peaks in the DNase1-seq data that are able to capture the dynamics of DNA accessibility for TF binding, we refer to this setup as *dynamic* features.

ChIP-seq features

ChIP-seq data for 123 TFs of the HepG2 cell line were downloaded from ENCODE, considering files processed by ENCODEs uniform processing pipeline. ChIP-seq read counts were measured in ChIP-seq peaks overlapping a 3 kb window defined around the gene's TSS (mapped against genome hg38) to be combined with the HLC/PHH single cell data for model training. We refer to these features as *ChIP-seq* features.

Figure 1 illustrates the genomic region in where the three feature setups (*static*, *dynamic*, and *ChIP-seq*) are generated.

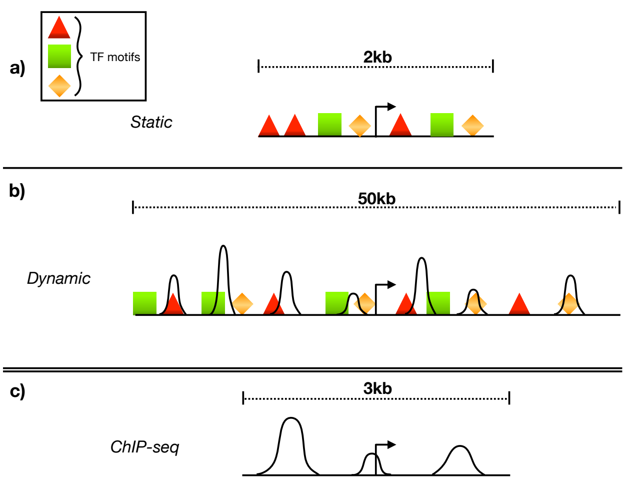

Figure 1. Genomic regions where the **(a)** *static*, **(b)** *dynamic*, and **(c)** *ChIP-seq* features are generated from.

Single cell RNA-seq data as response for the statistical models

We generated single cell RNA-seq data for 657 HLC differentiated from iPSC / PHH (HLC/PHH) as described in Supplementary Methods. These cells contain two annotated cell types, Primary Human Hepatocyte (PHH) and Hepatocyte Like Cells (HLC) with 288 and 369 cells, respectively. Gene expression is quantified in TPM (Transcripts Per Million). The expression values of all genes (TPM normalization) measured for a single cell, are considered as a task for the multi-task learning framework. The fastq files for this scRNA-seq data set is submitted to EGA and is accessible via EGAS00001004201 accession code.

In addition, we obtained the Human Skeletal Muscle Myoblasts (HSMM) data from [20]. It is worth mentioning that we only generated the *static* features for this data, as there was no valid annotation of the cells that we could rely on for the downstream analysis in our study. Therefore, this data set was only used to demonstrate the results based on the different choices of tree structures required for the tree-guided MTL models.

Imputation of single cell RNA-seq data

We used the scImpute method [21] on two single cell RNA-seq data sets. We set the parameter k to 2 for the HLC/PHH and 1 for the HSMM data. We use and compare the two variants of the gene expression data set, imputed and unimputed.

Filtering

We applied the filtering approach suggested by Monocle's tutorial [22] on the single cell RNA-seq data. At first, the detected genes were defined using the *detectGenes* function by setting the *min_expr* argument to 0.1. A gene is kept if there are at least 10 cells, in which the gene was detected (based on the aforementioned definition of detected genes), otherwise discarded.

We further reduced the gene set, by completely removing all the affinities computed for the genes that the variance in their feature space (TF affinities) was less than the third quartile of the variances measured for each gene. More precisely, given the F_S matrix, we compute the variance over the TF affinities for each genes, as follows:

$$var_i = variance(F_S[i,]),\ i \in \{1, \cdots, n\}, \tag{1}$$

where $F_S[i,]$ is a vector of size p, holding the affinity values in the ith row. Next, we define a threshold t based on the third quartile computed over var_i's $\forall i \in \{1, \cdots, n\}$, as a cutoff to decide whether the $gene_i$ should be kept or not:

$$gene_i : \begin{cases} \text{kept} & \text{if } var_i \geq t \\ \text{discarded} & \text{else}\,. \end{cases} \forall i \in \{1, \cdots, n\}, \tag{2}$$

In addition, we removed the TFs that their corresponding gene expression was zero.

Similarly, we applied these filtering steps on the other two feature setups, *dynamic* and *ChIP-seq*.

Statistical learning frameworks

Here, we describe two distinct statistical learning frameworks, single-task learning (*STL*) and multi-task learning (*MTL*). The *MTL* approach is further categorized into ordinary MTL (*OMTL*) and tree-guided MTL (*TRIANGULATE*).

We partitioned the data into training (60%) and test (40%) sets. 5-fold cross-validation was performed on the training set to select the best hyper-parameters for all models. The TF and gene expression data are normalized to have zero mean and unit variance. We use Pearson correlation computed between the predicted expression and measured expression values on the same test set to assess performance for all models.

Single-task learning method

We trained individual regression models with elastic net regularization through a 5-fold cross-validation model selection scheme, exploring the α parameter within the range of 0 and 1 with step size of 0.1 using the *glmnet* package in R [23].

Multi-task learning methods

Let $X \in \mathbb{R}^{n \times p}$ denote the input matrix for n observations (samples) and p features. Let $Y \in \mathbb{R}^{n \times k}$ denote the response matrix, whose columns are vectors of observations for k tasks. We look for an appropriate coefficient matrix, $B \in \mathbb{R}^{p \times k}$ that establishes the linear relation between X and Y with the error term ϵ as described in the following formula:

$$Y = XB + \epsilon\,. \tag{3}$$

There are various ways to obtain the optimal values for the B coefficient matrix. In this section, we describe several multi-task learning setups used in this study to understand the performance of different formulations and also downstream interpretation of the results.

Ordinary MTL

To optimize a multi-task regression model with elastic-net regularization, the following objective function is used:

$$B^* = arg\min_B(\Sigma_{i=1}^k(y_i - X\beta_i)^T.(y_i - X\beta_i) + \alpha\Sigma_{j=1}^p\|\beta^j\|_2), \quad (4)$$

where B^* denotes the optimal coefficient matrix, α is a tuning parameter that controls the magnitude of the coefficients through the L_2 norm regularization, and y_i is a vector of size n holding the response values of the ith task. β_i denotes the coefficients corresponding to the ith task (column) of matrix B. Similarly, β^j denotes the jth row of matrix B.

Given the optimization formula, we trained an MTL model with elastic net regularization using the R *glmnet* package [23], where the family argument was set to *mgaussian* to account for the multi-tasking nature of the setup. We used 5-fold cross validation to optimize over the α search grid defined within the range of 0 and 1 with the resolution of 0.05. The models generated using this formulation are referred to as ordinary MTL (OMTL) throughout the remaining of the text.

Tree-guided group-lasso MTL

In the ordinary MTL scenario all tasks share the same relevant features. However, it is possible that a subset of highly related tasks may share a common set of relevant features, whereas weakly related tasks are less likely to be affected by the same features. An improvement was proposed by [24] to address this shortcoming of OMTL models. Through their proposed method, which they refer to as tree-guided MTL, the relationship among the tasks is represented as a tree T with V vertices. Each leaf node of T is associated with a task and the internal nodes reflect the groupings of the tasks. This tree structure can be inferred directly from the data or may be available as prior knowledge beforehand. Within this tree, each node $v \in V$ is associated with a weight w_v, typically representing the depth of the subtree rooted at node v. The optimization formula for tree-guided MTL is:

$$B^* = arg\min_B(\Sigma_{i=1}^k(y_i - X\beta_i)^T.(y_i - X\beta_i) + \lambda\Sigma_{j=1}^p\Sigma_{v\in V}\|w_v\beta_{G_v}^j\|_2), \quad (5)$$

where λ is the regularization parameter and $\beta_{G_v}^j$ is a group of regression coefficients $\{\beta_i^j : i \in G_v\}$. We used the *LinearMTL* package implemented in R ([1]), to train the tree-guided MTL models. We first partitioned 60% of the data for training and 40% for test. Then, we normalized the data to zero mean and unit variance. For the purpose of model selection, we performed a 5-fold cross validation, through which 21 distinct values of λ, defined within the range of 0 and 1 with the resolution of 0.05, were explored. Finally, we trained the models by setting the maximum number of iterations to 1000.

Construction of trees used for the tree-guided MTL models

The gene expression matrix is used to infer the tree structure of the tree-guided MTL models. In order to assess the sanity of the models, we created a randomized gene expression matrix, to contrast the models trained on the real data with the random data.

We generated several trees derived from the gene expression data to guide the optimization of the tree-guided MTL models. Figure 2 summarizes the description of the tree structures listed below.

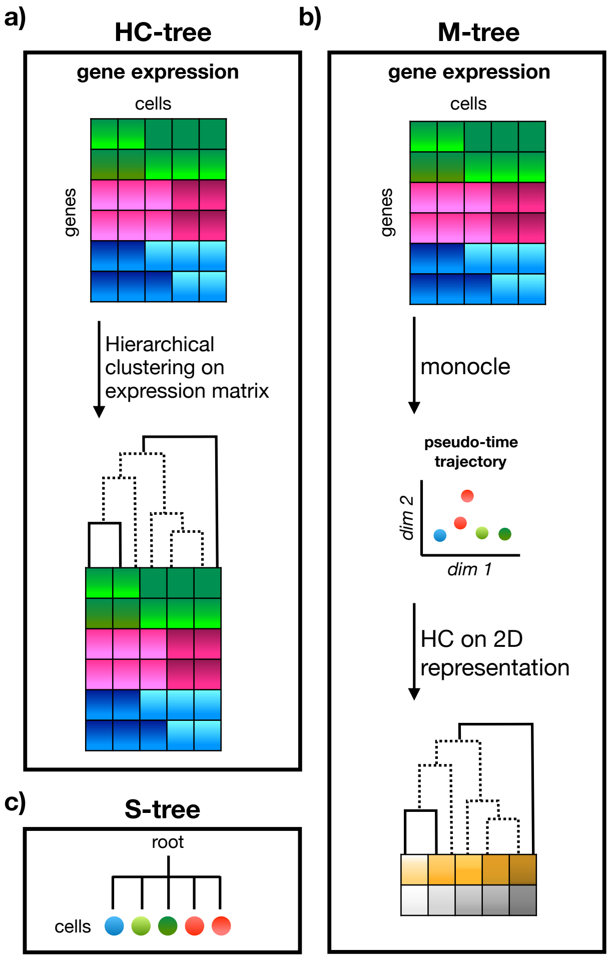

Figure 2. Schematic illustrations for the tree structures. **(a)** Performing hierarchical clustering on the gene expression matrix produces the *HC-tree*. **(b)** A pseudo-time trajectory is obtained using the Monocle software from the gene expression matrix. The resulting trajectory shown in a reduced 2-dimensional space is used to generate the *M-tree* structure. **(c)** Cells are connected to a root node forming a star shape tree, regarded as a baseline for the tree required models.

1 https://github.com/tohein/LinearMTL

- **HC-tree**: We used *BuildTreeHC* function of the *LinearMTL* package using the complete linkage and $1 - Pearson correlation$ as the dissimilarity measure to apply hierarchical clustering on the real single cell expression data. The clustering tree is then used to guide the tree-guided MTL models.
- **M-tree**: Coordinates of the single cells in the reduced dimension space (a matrix of size $2\times$ number of cells) derived from the Monocle model trained on the real data. Using the *BuildTreeHC* function, as described in *HC-tree*, we generated the tree structure from the coordinates in the reduced dimension space.
- **S-tree**: We constructed another tree that serves as a baseline for our tree-guided MTL models. The tree structure forms a star shape, with a root and as many children nodes as the number of cells. More precisely, let k be the number of cells. Then, the star tree has $k + 1$ nodes, labeled by $0, 1, \cdots, k$, where 0 represents the root and the remaining nodes represent the k cells. Every non-root node has one and only one edge connecting it to the root. Clearly, the root has immediate links to other nodes, i.e., degree of k. This tree structure is considered baseline, because it does not suggest any particular grouping of the cells, as they all are uniformly connected to the root.
- **R-tree**: In order to generate appropriate random data, we shuffled the gene expression profile of each single cell. Given that the genes are arranged in rows and cell in columns, for each column, we shuffled the gene expression values across the genes, and then used this shuffled gene expression matrix as the input to the Monocle tool. Finally, we trained the tree-guided MTL model using the *M-tree* structure described above. Replacing the gene expression matrix in Figure 2b with the shuffled expression matrix produces the *R-tree* setup.

The implementation of our workflow can be accessed via the github repository: https://github.com/SchulzLab/Triangulate.

Selection for heat map visualization

Since for the *static* features, several hundreds of TFs were included in the set and visualizing this many TFs makes the interpretation difficult, we decided to shrink this set by selecting those that pass a certain criteria. Essentially, for a given TF arranged in the rows of the coefficient matrix, we compute the sum of absolute regression coefficients for that TF across all cells. If this value is higher than our predefined threshold of 0.5, we keep that TF, or discard it otherwise, for visualization of *TRIANGULATE* results in the heat map in 6.

Correlation analysis between expression and inferred TF activity

We define the *TF-expr-cor* as the Spearman correlation between the cell-specific expression of a TF and the inferred TF activity per cell (coefficients) of the *TRIANGULATE* model. We used a permutation approach to obtain a significance estimate for the correlation values.

First, we used the *R-tree* model, which is based on permuted expression values, to obtain an estimate of the regression matrix $\tilde{B}$. Second, we compute the Spearman correlation values between the permuted expression values and the computed regression coefficients in $\tilde{B}$ for each TF over all cells. This defines the null distribution of the *TF-expr-cor* values. In other words the *TF-expr-cor* values using the *R-tree* model define the null model.

This we can compare to an actual model. For example, using the *HC-tree* model, we computed the *TF-expr-cor* values. For our analysis (e.g. Fig. 8) we removed those values that lied within the range of *TF-expr-cor* values under the null model (Suppl. Figure 1).

To illustrate the interesting TFs only, we further reduced the TF set by keeping those where the sign of correlation agreed with the sign of sum of coefficients across cells for a given TF.

$$TF_i : \begin{cases} \text{kept} & \text{if } Expr(TF_i) \times \Sigma_j B_i^j > 0 \\ \text{discarded} & \text{else}. \end{cases} \quad \forall i \in \{1, \cdots, n\}, \quad (6)$$

where, the function $Expr(TF_i)$ denotes the log2-transformed TPM values of TF_i measured in single cells, B_i^j is the coefficient corresponding to TF_i in cell j, and n is the number of TFs.

Running SCENIC

Single-CEll regulatory Network Inference and Clustering [11] was performed using the python implementation pySCENIC ([2]) based on human TF ranking data base version 9 (motifs-v9-nr.hgnc-m0.001-o0.0 [3]) and human motif to TF annotation downloaded from [4] (v9) as outlined in the package manual.

Results

We study the cell-specific association of regulatory elements by coupling distinct TF feature data with measurements of gene expression in single cells. For this purpose, we designed three different feature setups (*static*, *dynamic*, and *ChIP-seq*) representing the TF binding scores in various genomic regions (see Materials and Methods, and Figure 1). In order to conduct a supervised regression framework, we exploited three single cell gene expression data sets (HLC/PHH, HSMM, and T cell) as the response variable. We performed several filtering steps to remove the poor quality data as described in Materials and Methods. After discarding these low quality values from the HLC/PHH data, 238 cells were remained with 14142, 4827, and 14188 genes for *static*, *dynamic*, and *ChIP-seq* features, respectively. Similarly, for HSMM, the reduced data set contained 18,402 genes and 297 cells for the *static* features.

Given this data, we were able to train our models in two ways, as single tasks or combined with multi-tasking. As illustrated in Figure 3a, in the single task learning case, each cell provides the response vector for an individual optimization problem solved through an elastic net regularization (see Materials and Methods). Therefore, the total number of statistical models needed to be generated is equal to the number of cells in the given gene expression data set. On the other hand, when the multi task learning approach is used, the complete gene expression matrix is regarded as the response variable, where one model is created in the end. In this scenario, model coefficients are represented by a 2-dimensional matrix B, where each entry of B reflects the inferred activity of a particular TF in a specific cell.

2 https://github.com/aertslab/pySCENIC, v0.9.19
3 https://rdrr.io/github/aertslab/RcisTarget/man/motifAnnotations.html
4 https://resources.aertslab.org/cistarget

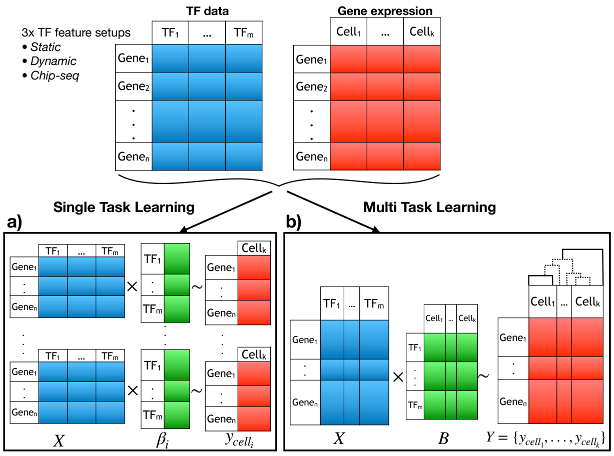

Figure 3. Schematic illustration of the learning setups, single **(a)** and multi **(b)** task learning. Common input files consisting of TF data (either, *static*, *dynamic*, or *ChIP-seq*) and single cell gene expression are provided for both learning schemes. The rows of the feature matrix, X, are the genes for which one of the feature setups described previously would be used. The response matrix, Y, consists of the gene expression values measured in single cells. And finally, the coefficients matrix, B, establishes a linear association between the X and Y, where the rows indicate the features and columns the cells.

Tree-based models generally result in better performance

The tree-guided MTL models expect a tree structure to guide the model on how the tasks should be grouped when optimizing the objective function. However, the choice of the tree for the tree-guided MTL can be arbitrary as this tree is considered as a hyper-parameter set by the user. Therefore, we explored several trees for which we presumed they can represent the structure existing in the single cell gene expression data.

As a trivial and straightforward choice, we applied hierarchical clustering directly on the gene expression data. The tree obtained from the hierarchical clustering was then used to train the tree-guided MTL models, which we refer to as *HC-tree*. The next intuitive choice was to infer the tree structure from the pseudo-time ordering applied on the single cells, as the differentiating cells should be placed closer to each other in this trajectory. Using the Monocle [20] tool (version 2), we were able to construct this trajectory for the single cell expression data. Through traversing the trajectory obtained from Monocle, we built a tree representing the pseudo-time ordering of the cells. Since the transformation from the pseudo-time ordering to a tree can be arbitrary, we applied hierarchical clustering on the matrix holding the data for pseudo-time ordering and used the resulting tree for our tree-guided MTL models. We refer to this tree structure as *M-tree* (see Materials and Methods).

We further examined the performance of the tree-guided models with two other types of tree structures, *random* and *star* (see Materials and Methods). We used *S-tree* as a baseline for the tree-guided models, as this structure imposes a uniform clustering of the cells (they all are at the same level relative to each other). Also, we introduced the *R-tree* to compare the performance of the models trained on the true data with the random data. Figure 4 compares the performance of *R-tree* and *S-tree* with the *HC-tree* and *M-tree* models. These results suggest that the choice of hierarchical tree, performed on either the full gene expression data or the reduced space, are valid and reliable as they outperformed the *R-tree* and *S-tree* models.

Apart from the tree-guided models, we also generated the ordinary multi-task learning (*OMTL*) models to examine the efficiency of the tree-guided over *OMTL* models. Scatter plots provided in Supplementary Figures 2 and 3 allow us to compare the performance of *OMTL* models with the tree-guided MTL models, where different trees are used.

In addition to the *OMTL* models, we trained individual single-task models, by providing the gene expression profile per cell as the response variable of each model (see Materials and Methods). The predictions obtained from each individual model were later used to compute the correlation values between the prediction and actual measurements of gene expression.

Figures 4 illustrate the distribution of Pearson correlation coefficients calculated between the predicted and measured values of gene expression for all the tree-guided multi-task learning models (*HC-tree*, *S-tree*, *M-tree* and *R-tree*) as well as the *OMTL* and *STL* models, for both data sets.

The cell-wise comparison of all statistical models are provided as scatter plots in Supplementary Figures 2 and 3. These results indicate that PHH cells are, in general, more difficult for the model to predict their gene expression, irrespective of the statistical model used. However, it is interesting to see that the *STL* models tend to suffer more compared to the tree-guided MTL or the *OMTL* models, where they are essentially able to benefit from the information sharing among the tasks.

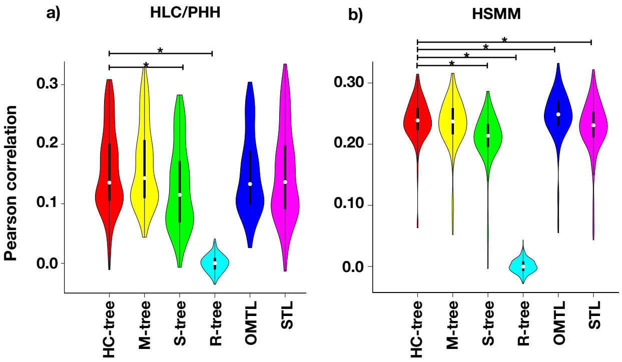

Figure 4. Comparison of MTL and STL models (shown on x-axis) on HLC/PHH **(a)** and HSMM **(b)** data sets. The y-axis is the Pearson correlation coefficient computed between the predicted and measured gene expression values on the test data. Two sided Mann-Whitney (unpaired) test with significance cutoff of 0.05 was performed between *HC-tree* and the other models. Pairs with significant differences are marked with an asterisk. See Supplementary Figure 4 for a complete comparison of p-values on all pairs of models.

Given that the *HC-tree* and *M-tree* result in better performing models in both HSMM and HLC/PHH data sets, we decided to pick either of these methods to proceed with the rest of our analysis. However, due to the computational burden incurred by the additional step of running the Monocle software for inferring the trajectory in the *M-tree* case, we favored *HC-tree* over *M-tree*. Therefore, the following results presented in this manuscript are obtained from the *HC-tree* structure, which we call TRIANGULATE in the remainder of this manuscript.

The impact of the number of cells on prediction results

As the number of cells in the data set can play a crucial role in a multi-task learning setup, similar to what we proposed, we decided to challenge our *TRIANGULATE* by training it on fewer number of cells using the static features. Therefore, we down-sampled the HLC/PHH cells using 5 different percentages of cells (50%, 40%, 30%, 20%, and 10%). Table 1 shows the average number of detected genes (the number of genes having expression of at least 1 in a given single cell) used for training the *TRIANGULATE* models. This table also provides the Pearson cor-

Table 1. Number of detected genes (mean ± sd) and Pearson correlation coefficients (mean ± sd) on test data for down-sampled HLC/PHH cells based on 10 – 50% of the whole data. Results obtained from the static features.

% of data	# of detected genes	correlation
10	4,522 ± 1903	0.10± 0.04
20	4,545 ± 1820	0.12± 0.05
30	5,025 ± 1768	0.13± 0.06
40	4,960 ± 2000	0.15± 0.07
50	4,818 ± 1781	0.15± 0.07

relation measured between predicted and measured single cell gene expression on test data of these down-sampled HLC/PHH cells.

Overall, we observe that *TRIANGULATE* shows a robust performance when the number of cells are varied. However, there is an apparent trend of performance loss as the number of cells are decreased.

The impact of feature types on the prediction results

We wanted to explore the associations of gene expression in single cells to features that are independent of the cell content or configuration. Therefore, we designed a feature setup, which we named *static*, to link the cis-regulatory characteristics of ∼ 700 transcription factors with the gene expression measurements of single cells (see Materials and Methods).

Figure 1 schematically illustrates the genomic area, where the *static*, *dynamic*, and *ChIP-seq* features are generated from. In *static* features, for each transcription start site of a gene, the TF binding affinities are measured within the 2 kb window around the TSS. These affinity scores are used to form the feature matrix for the *static* setup (Figure 2a). Figure 2b, illustrates the *dynamic* setup, where peaks are obtained from DNase1-seq data and used to identify the open chromatin regions in a 50 kb window around the TSS. The TF binding affinities are computed in the segments of this 50 kb window that correspond to the peaks. Finally, Figure 2c, shows the region in where the reads of ChIP-seq data of 123 TFs are counted. The resulting measurements form the *ChIP-seq* features.

Figure 5 describes the performance of the *HC-tree* MTL approach on the three features setups for the HLC/PHH data set. We observed that TF features derived from measurements in HepG2 cells (*dynamic* or *ChIP-seq* features) showed better performance than the *static* feature setup. Presumably, this reflects a more liver-specific association between a TF's binding affinity and chromatin openness around a gene in those setups. Overall, the ChIP-seq features led to the most accurate models.

Imputation generally improves the accuracy

Single cell data suffers from inherent technical noise of so called dropouts. Dropouts are referred to genes that are falsely identified as zero-expressed. In simpler words, any zero that is observed in the expression count matrix of single cell data, can be viewed as either correctly or incorrectly identified as a silent gene due to the dropout effect. There have been several methods, *e.g.* [25, 21, 26], that attempted to address this problem by imputing the missing expression values, but each of these methods has its own assumptions.

The results shown in previous sections are performed on the original unaltered expression data. However, we were curious to find out how the results will change when we impute missing values potentially introduced by the dropout effect. Therefore, we imputed missing data using the scImpute tool ([21],

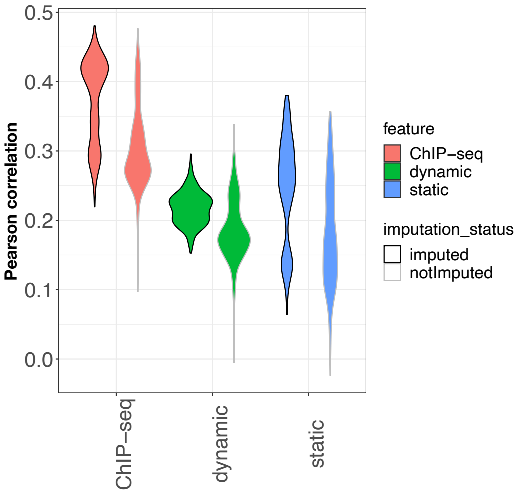

Figure 5. Comparison of different feature sets using the *HC-tree* MTL structure on the test partition of the HLC/PHH data (Pearson correlation, y-axis). *Dynamic*, *ChIP-seq*, and *static* feature setups are colored with red, green, and blue, respectively. The imputation status is indicated by different border colors, black for imputed (left) and gray for not imputed (right) for each feature set.

see Methods) and repeated the experiments described in with the difference of using the imputed expression values as the response matrix.

Figure 5 provides an overview of the performance of the *HC-tree* MTL model on the three feature setups, comparing the use of original or imputed scRNA-seq counts. These results reveal that the imputation enhances the prediction accuracy, regardless of the feature setup. It is interesting to observe that for the *dynamic* setup, not only the correlation values are increased, but also the distribution of these values is changed in favor of having a smaller variance across the cells. The change of distribution is notable for the other two setups as well, but that does not necessarily lead to a smaller variance.

Cell type specific TF activities inferred from the model coefficients

Observing such difference in the prediction accuracy, inspired us to inspect the model coefficients that correspond to the activity of TFs in cells. The heat map in Figure 6 depicts the coefficients of the top features (see Materials and Methods) derived from the HC-tree model trained on the *static* features to predict the gene expression in HLC/PHH cells.

In this heat map, it can be noted that, firstly, the cells are clearly clustered according to the model coefficients, separating the HLC and PHH cell types. Secondly, these results show certain groups of TFs playing distinct roles in regulating gene expression in cell sub populations. For instance, the transcription factor YY2 holds positive coefficient values for the HLC cells, whereas its values for the PHH cells are negative. This is interesting, because YY2 may perform a dual affect on gene expression, *i.e.*, it can both repress and activate transcription [27].

On the other hand, HNF1A, which is essential for the expression of various liver-specific genes, was considered less relevant for the HLC cells by the model, as it has mostly assigned zero to coefficients corresponding to HNF1A. However, HNF1A holds positive coefficient values for the PHH cells. GMEB1 is another factor that shows a variable activity between the two cell types. Data from the *Human Protein Atlas* suggests it to be a prognostic marker in liver cancer (https://www.proteinatlas.org/ENSG00000162419-GMEB1).

In addition, we observe that PBX3 and MAFG are TFs that seem to be active for both cell types according to our model. It has been shown that PBX3 plays a crucial role in the transcriptional program of human liver tumor-initiating cells [28]. Liu and co-workers [29] observed that MAFG is positively correlated with the progression of tumor cells especially in cholangiocarcinoma and hepatocellular carcinoma patients.

We additionally inspected the model coefficients to check if the two cell types were still separable on the down-sampled data sets introduced before (Table 1). Supplementary Figure 5 shows that *TRIANGULATE* was able to assign the coefficients of the model appropriately according to the cell types in the down-sampled data.

Comparison of results with SCENIC

As SCENIC [11] is also able to produce cell-specific TF activity scores, we were interested in comparing the results of our models with the AUC values that SCENIC computes to represent the TF activity (see Materials and Methods). Since the AUC values are positive, SCENIC can only infer positive associations, in contrast to our method that is able to deduce negative associations as well. As a result, comparing the activity matrix directly (Suppl. Figure 6), was not a meaningful approach.

For this purpose, we defined per TF activities by adding up their activity scores (AUC values for SCENIC and scaled coefficient values for *TRIANGULATE*) across cells. The top 20 active TFs obtained from each approach were compared with a set of known liver-specific TFs previously collected through literature search [18]. The number of overlapping TFs between the liver-specific set and top 20 active TFs obtained from *TRIANGULATE* is shown in Figure 7a (similarly for SCENIC in Figure 7b). These results indicate that *TRIANGULATE* is able to identify more liver-specific TFs than SCENIC among the top 20 active TFs.

We further noticed that YY1, TBP, HNF4G, KLF, and CEBPA were the liver-specific TFs that only *TRIANGULATE* was able to identify among its top 20 TFs and SCENIC could not.

We were also interested in identifying the TFs that showed a significant difference in their inferred activity between the HLC and PHH cell types. We applied a (two-sided) Mann-Whitney test using the significance cutoff of 0.1 on the multiple-testing corrected p-values (*Benjamini-Hochberg* method) to select the TFs that are significantly different between the HLC and PHH cells. In our proposed method, this test was applied on the model coefficients and in SCENIC on the AUC values obtained for the HLC/PHH cells. The Venn diagram shown in Supplementary Figure 7 illustrates the number of TFs common between the liver-specific TF set, SCENIC, and the *TRIANGULATE* approach. It can be seen that *TRIANGULATE* and SCENIC have 26 and 28 TFs in common with known liver-specific TFs, respectively. This indicates that our approach is able to find liver-specific TFs and mostly agrees with the results of SCENIC when considering differences in regulation between the two cell types.

The agreement we observed between the liver-specific TFs and top active TFs suggested by SCENIC and *TRIANGULATE* inspired us to advance our investigation by analyzing the inferred TF activities in each individual cell.

Using the pseudo-time ordering of the cells obtained from *monocle*, we displayed the cells in the 2-dimensional trajectory space and marked each cell based on their inferred activity obtained from SCENIC or *TRIANGULATE* as well as the expression of the TF in single cells (Suppl. Fig. 8 and Fig.7c). It should be noted that the scores SCENIC obtains are a function of a TF's gene expression, which is used in the first step of the method. Thus, it is not surprising to see that the SCENIC AUC scores often agree well with the trend observed in TF expression values, but rather confirms that the approach works as intended. As shown in Fig. 7c, YY1 appears to be expressed across the single cell trajectory. This behaviour is reflected in the inferred TF activities by SCENIC and *TRIANGULATE*. However, as mentioned earlier, this TF did not appear among the top 20 active TFs for SCENIC. This is due to the very small AUC values that SCENIC computed for YY1 (maximum of 0.07).

Another interesting example is TFDP1. We observed that *TRIANGULATE* assigned negative coefficients to the PHH cells of TFDP1 (Figures 6 and 7c). It has been shown that TFDP1 together with E2F1 are involved in regulating hepatocellular carcinoma cells through a knockdown experiment that confirmed a physical interaction of KPNA2 with E2F1 and TFDP1 [30].

We additionally found that ARID3A is among the TFs that had negative activity consistently across the single cells. We found evidence by literature that ARID3A is identified as a repressor in embryonic regulation [31, 32].

From the trajectory plots provided in Figure 7c, it can be seen that the activities of TFDP1 and ARID3A are concentrated on different subsets of cells. As previously mentioned SCENIC cannot infer negative TF activity, which can explain the small AUC scores (maximum of 0.27) assigned to the cells for these two TFs.

It is worth mentioning that the coefficient values we obtained from *TRIANGULATE* are in general very small. This is due to the large number of features each data point has. Since our model exploits ~ 700 TFs in its feature space, among which many are non-zero (selected by the model), the values assigned to these coefficients need to be small so that the linear combination of the coefficients and feature data falls within the range of response values. Since we want to explore the negative associations, we show the original coefficients in Fig. 7.

To further evaluate and compare the performance of *TRIANGULATE* with SCENIC, we acquired another data set of single cell gene expression (Supplementary methods). This data consists of the CD4+ T cells from a liver cancer patient (accession number GSM2602298) [33]. 176 cells are annotated as normal and 230 cells are annotated as tumor. We ran SCENIC and *TRIANGULATE* in the same way as described earlier to identify the top 20 active TFs. We also prepared a list of T cell-specific TFs from literature provided in Supplementary Table 1. By overlapping the set of known TFs regulating T cells with the top 20 active TFs obtained from *TRIANGULATE* and SCENIC, we were able to find 15 and 8 TFs, respectively. The Venn diagrams shown in Supplementary Figure 9 illustrate this overlap for both approaches. Similar to the analysis of the HLC/PHH data set, *TRIANGULATE* was able to find more cell-type specific TFs than SCENIC.

TF expression and its inferred activity

As we often observed agreement between the expression and *TRIANGULATE* coefficients (scores), we decided to systematically assess this similarity. To inspect how much the inferred TF activities agree with the expression of a TF's gene in single cells, we designed a correlation analysis to evaluate the similarity between these two quantities. As described in Materials and Methods, we introduce *TF-expr-cor*, the Spearman correlation between the coefficients obtained from the *TRIANGULATE* model and the log2-transformed TPM values representing the expression of TFs. Figure 8 illustrates the *TF-expr-cor* values for a subset of TFs that had higher values than a null model (the *R-tree* model). According to this analysis, ARID3A and KLF6 have the smallest negative and largest positive *TF-expr-cor* values, respectively.

Contrasting the gene expression signal of ARID3A shown

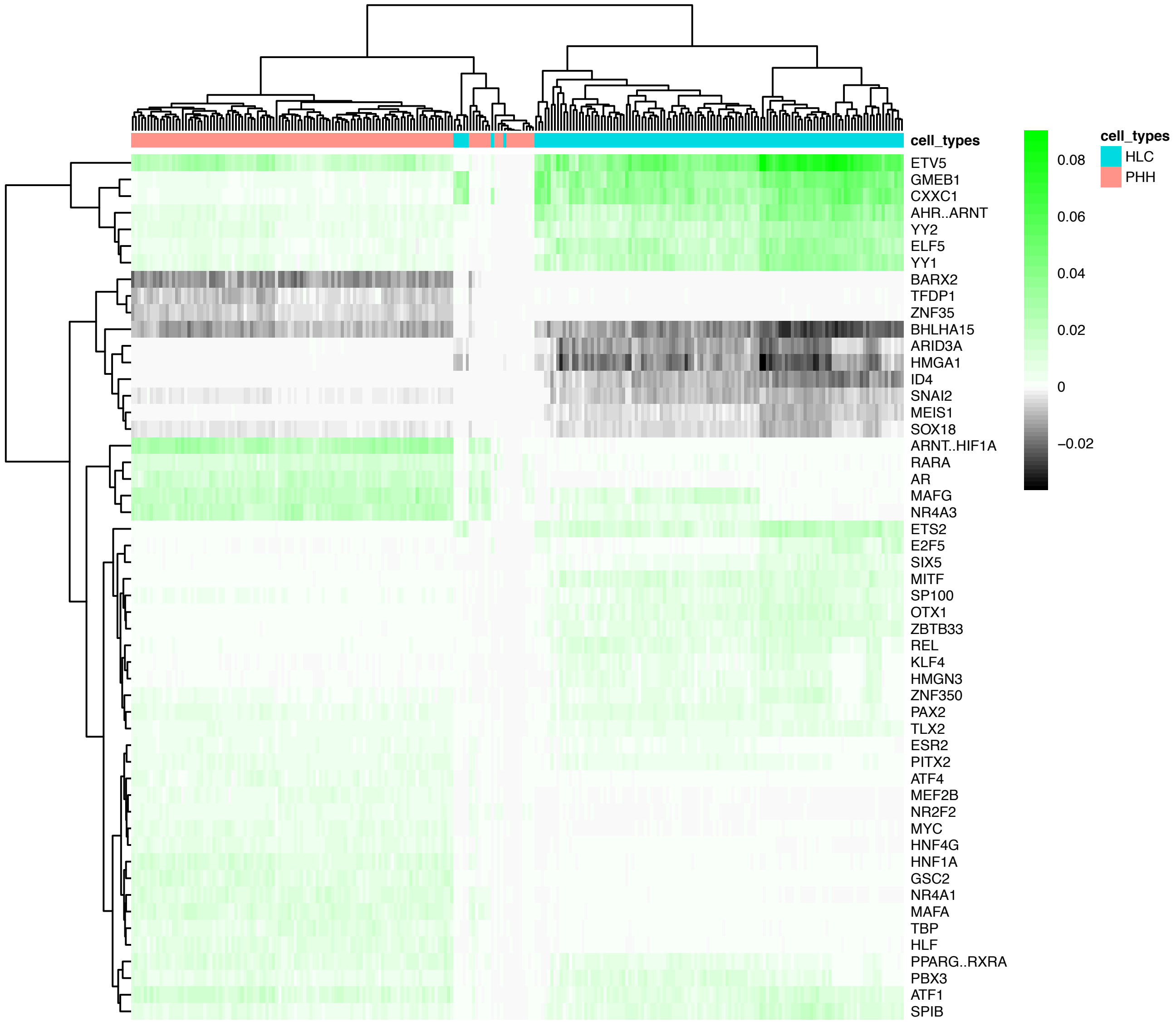

Figure 6. Coefficients of the tree-guided MTL model using the *HC-tree* structure. Heat map illustrating the top features (see Materials and Methods) derived from the tree-guided MTL trained on the *static* features to predict the unimputed gene expression in HLC/PHH cells.

in Figure 7c with the inferred activity from *TRIANGULATE* delineates the strong negative correlation between the gene expression of ARID3A and its *TRIANGULATE* coefficients that we observed in Figure 8.

For the TFs where their correlation values were relatively high, we also observed a less sparse signal (fewer zeroes) in their corresponding gene expression data. This observation holds for both positive and negative correlations. However, one should not expect high correlations for all TFs with high inferred activities. Still, if both quantities correlate this may point to regulators of interest concerning the studied data set.

Discussion

The discrepancies observed among the gene expression profiles in single cells, trivially, hint at the existence of specific differences in the transcriptional regulatory program. Devising computational methods that are able to infer associations between gene expression in single cells and cis-regulatory motifs, as well as epigenetic characteristics, has attracted the attention of researchers in the field, *e.g.* [8, 11, 10, 9].

In this work we analyzed a regression formulation, where TF features based on sequence motif matches, bulk ChIP-seq peaks or DNase1-seq peaks, were used to predict gene expression in individual cells. Previously, such regression formulations were only done in the context of bulk gene expression prediction. Obviously, for single cell data this is a harder problem, as most of the single cell data available is very sparse with only few read counts per gene, if any. These technical limitations are challenging to address.

On the two data sets we investigated, we found that the correlation on test data, is not overwhelming, but it is hard to come up with an expected correlation. Using similar features in a regression of bulk RNA-seq, coupled with epigenomic data sets, led to correlation coefficient values of ~ 0.3 to 0.6 [18], but with Dnase1-seq and RNA-seq data obtained from the same cells.

We wondered if imputation would improve the correlation on the test set. For that, we generated our statistical models on the imputed gene expression data. The results displayed in Figure 5 consistently show an improvement in accuracy for the imputed models. We think that by imputing the missing values of gene expression, a stronger connection may have been established between the TF and gene expression data. Therefore, the signal existing in the features (TF data) better reflects

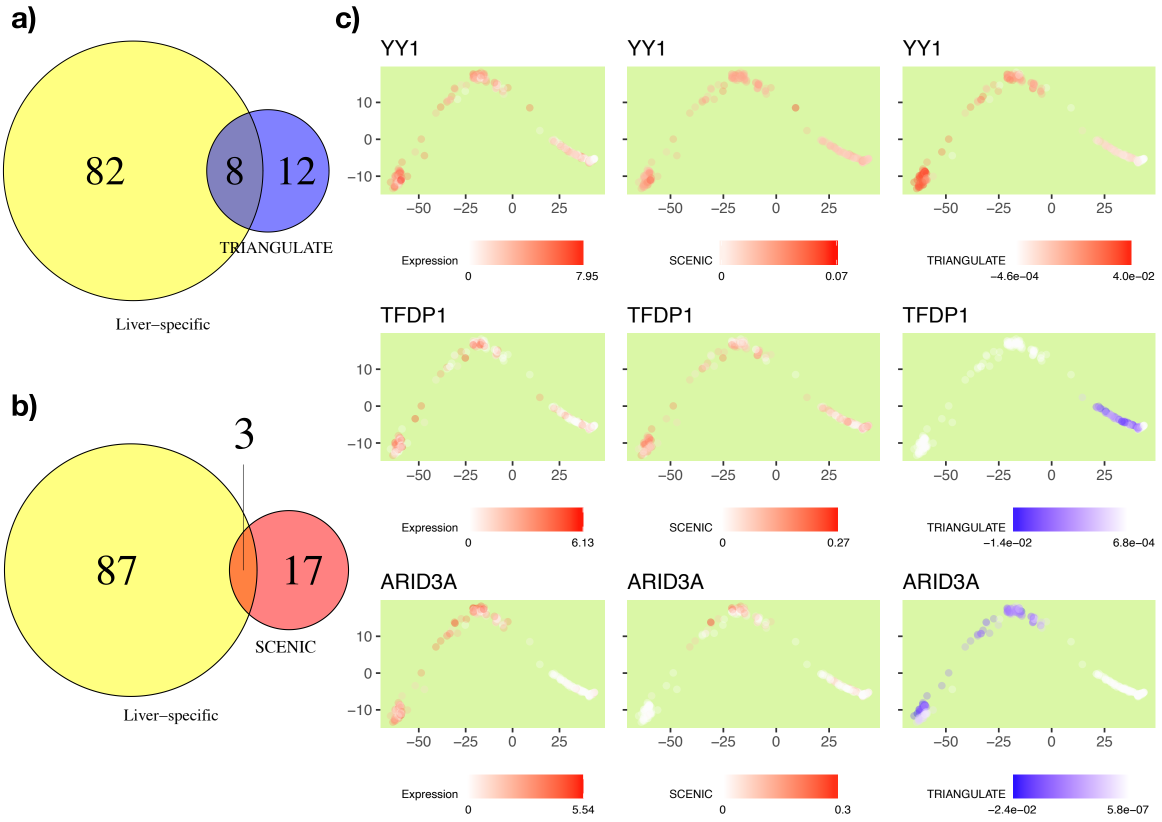

Figure 7. Comparison of inferred TF activities. Venn diagram depicting the number of overlapping TFs between liver-specific and each of **(a)** SCENIC and **(b)** *TRIANGULATE* models. The top 20 TFs that were identified to be active are used for the comparison of SCENIC and *TRIANGULATE*. **(c)** Exemplary TF trajectories are shown in the single cell trajectory based on the Expression of TF (left), the AUC score obtained from SCENIC (middle), or the coefficients of the *TRIANGULATE* model (right).

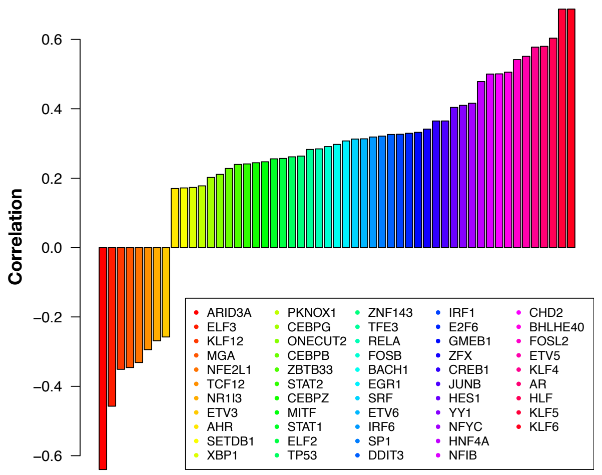

Figure 8. Spearman correlation between a TF's gene expression and inferred TF activity from *TRIANGULATE* over all cells in the HLC/PHH data set. TFs are sorted by correlation and correlation values smaller than obtained by a permutation analysis are not shown (see Methods).

the variances in the response (gene expression).

We designed a general framework for establishing cell specific associations through employing tree-guided multi-task learning [24]. This framework benefits both from the information sharing delivered by multi-tasking and also grouping the cells according to the tree structure provided as an additional input. The choice of the tree is a hyper-parameter (user-dependent). We tried exploring different tree structures, and our findings indicate that the performance of the models is influenced by this choice. In addition, we see that depending on the data set, the best performing model is generated from different tree structures. Therefore, it is difficult to suggest an all-purpose tree inference approach that would work best for all data sets. Besides, we think that the optimization model could be modified such that it is not restricted to a tree structure but an arbitrary graph for grouping the tasks (cells).

Another area for improvement is the distribution assumption. The Gaussian distribution used in the current optimization does not best address the specifics of count data, nor the single cell measurement noise such as dropouts. As a future work, we propose incorporating the negative binomial distribution to better account for these issues.

To check if the accuracy of our models would improve when a non-linear learning setup is applied, we designed a vanilla artificial neural network (NN, Supplementary methods). By comparing the median of the correlation between our linear models and this neural network, as shown in Supplementary Figure 10, we did not observe any significant improvement achieved by the NN models. Thus, another direction would be to adapt more sophisticated NN architectures, such as [5], to single cell data.

An obvious advantage of the linear modelling approaches is the straight-forward interpretation of TF regression coefficients. By inspecting the coefficients of our models, we were able to pinpoint distinct TFs that show cell type-specific regulation in HLC/PHH cells and showed that many liver-specific regulators could be inferred in this way.

Conclusion

The problem of identifying cell-specific regulatory elements is a difficult task, mainly due to the technical noise in the single cell data. However, in this study, we built several statistical models that lead to stable feature selection, which allows interpretable results. Also, it can be used directly to incorporate various approaches for designing TF features, such as transcription factor binding affinities and ChIP-seq signals.

As a future work, it would be interesting to extend this study using paired scRNA and single cell open-chromatin data [34, 35, 36], particularly for the design of *dynamic* features. Using this type of data allows us to estimate the TF activity in accessible chromatin regions defined based on individual single cells.

Availability of source code and requirements

Lists the following:

- Project name: TRIANGULATE
- Project home page: https://github.com/SchulzLab/Triangulate
- Operating system(s): x86_64-pc-linux-gnu (64-bit)
- Programming language: R (version >= 3.4.4)
- Other requirements: monocle(2.9.0), stringr(1.4.0), LinearMTL(0.2.0), doParallel(1.0.15)
- License: MIT

Availability of supporting data and materials

Preprocessed feature and expression matrices will be uploaded to GigaDB.

Declarations

List of abbreviations

HLC: hepatocyte like cells; HSMM: human skeletal muscle myoblast; MTL: multi-task learning; NN: neural network; OMTL: ordinary multi-task learning; PHH: primary human hepatocyte; STL: single task learning; TF: transcription factor; TPM: transcripts per million; TRIANGULATE: tree guided estimation of single cell regulation; TSS: transcription start site.

Ethical Approval (optional)

Not applicable.

Consent for publication

Not applicable.

Competing Interests

The authors declare that they have no competing interests.

Funding

This work has been supported by the DZHK (German Centre for Cardiovascular Research, 81Z0200101) and the DFG Clusters of Excellence on Multimodal Computing and Interaction [EXC248] and Cardio-Pulmonary Institute (CPI) [EXC 2026], and the DFG SFB/TRR 267 Noncoding RNAs in the cardiovascular system.

Author's Contributions

FBA and MS conceived, designed the project. FBA implemented the *TRIANGULATE* workflow and produced all figures/plots, FS generated the TF feature data, KK ran SCENIC on the HLC/PHH and T cell data sets, FBA analyzed the SCENIC results and produced the *TF-expr-cor* analysis, TH implemented the linearMTL package, DF, GG, KL, PN, JH and JW were involved in generation and/or primary processing of the HLC/PHH single cell gene expression data. FBA and MS wrote the draft manuscript. All authors approved the final the manuscript.

References

1. Liao JC, Boscolo R, Yang YL, Tran LM, Sabatti C, Roychowdhury VP. Network component analysis: Reconstruction of regulatory signals in biological systems. Proceedings of the National Academy of Sciences 2003;100(26):15522–15527. https://www.pnas.org/content/100/26/15522.
2. Schacht T, Oswald M, Eils R, Eichmüller SB, König R. Estimating the activity of transcription factors by the effect on their target genes. Bioinformatics 2014 08;30(17):i401–i407. https://doi.org/10.1093/bioinformatics/btu446.
3. Balwierz PJ, Pachkov M, Arnold P, Gruber AJ, Zavolan M, van Nimwegen E. ISMARA: automated modeling of genomic signals as a democracy of regulatory motifs. Genome research 2014 may;24(5):869–84. http://www.pubmedcentral.nih.gov/articlerender.fcgi?artid=4009616{&}tool=pmcentrez{&}rendertype=abstract.
4. Schmidt F, Schulz MH. On the problem of confounders in modeling gene expression. Bioinformatics 2018 08;35(4):711–719.
5. Zeng W, Wang Y, Jiang R. Integrating distal and proximal information to predict gene expression via a densely connected convolutional neural network. Bioinformatics 2019 07;36(2):496–503. https://doi.org/10.1093/bioinformatics/btz562.
6. Schmidt F, Kern F, Schulz MH. Integrative prediction of gene expression with chromatin accessibility and conformation data. Epigenetics and Chromatin 2020 02;p. in press.
7. Fiers MWEJ, Minnoye L, Aibar S, Bravo González-Blas C, Kalender Atak Z, Aerts S. Mapping gene regulatory networks from single-cell omics data. Briefings in Functional Genomics 2018 01;17(4):246–254. https://doi.org/10.1093/bfgp/elx046.
8. Mohammadi S, Ravindra V, Gleich DF, Grama A. A geometric approach to characterize the functional identity of single cells. Nature Communications 2018;9(1):1516. https://doi.org/10.1038/s41467-018-03933-2.
9. Ding J, Aronow BJ, Kaminski N, Kitzmiller J, Whitsett JA, Bar-Joseph Z. Reconstructing differentiation networks and their regulation from time series single-cell expression data. Genome Research 2018;28(3):383–395. http://genome.cshlp.org/content/28/3/383.abstract.
10. Yuan Y, Bar-Joseph Z. Deep learning for inferring gene relationships from single-cell expression data. Proceedings of the National Academy of Sciences 2019;116(52):27151–27158.
11. Aibar S, González-Blas CB, Moerman T, Huynh-Thu VA, Imrichova H, Hulselmans G, et al. SCENIC: single-cell regulatory network inference and clustering. Nature Methods 2017;14. https://doi.org/10.1038/nmeth.4463.

12. Suo S, Zhu Q, Saadatpour A, Fei L, Guo G, Yuan GC. Revealing the Critical Regulators of Cell Identity in the Mouse Cell Atlas. Cell Reports 2018 Nov;25(6):1436–1445.e3. https://doi.org/10.1016/j.celrep.2018.10.045.
13. Setty M, Helmy K, Khan AA, Silber J, Arvey A, Neezen F, et al. Inferring transcriptional and microRNA-mediated regulatory programs in glioblastoma. Molecular Systems Biology 2012;8(1):605. https://www.embopress.org/doi/abs/10.1038/msb.2012.37.
14. Lee S, Xing EP. Leveraging input and output structures for joint mapping of epistatic and marginal eQTLs. Bioinformatics 2012 06;28(12):i137–i146. https://doi.org/10.1093/bioinformatics/bts227.
15. Jain S, Gitter A, Bar-Joseph Z. Multitask learning of signaling and regulatory networks with application to studying human response to flu. PLoS computational biology 2014 dec;10(12):e1003943–e1003943. https://www.ncbi.nlm.nih.gov/pubmed/25522349https://www.ncbi.nlm.nih.gov/pmc/articles/PMC4270428/.
16. Dehghani Amirabad A, Schulz MH. Multitask regression for condition-specific prioritization of miRNA targets in transcripts. PeerJ Preprints 2016 Aug;4:e2377v2. https://doi.org/10.7287/peerj.preprints.2377v2.
17. Roider HG, et al. Predicting trancription factor affinities to DNA from a biophysical model. Bioinformatics 2007 Jan;23(2):134–141.
18. Schmidt F, Gasparoni N, Gasparoni G, Gianmoena K, Cadenas C, Polansky JK, et al. Combining transcription factor binding affinities with open-chromatin data for accurate gene expression prediction. Nucleic acids research 2017 Jan;45(1):54–66.
19. Schmidt F, Kern F, Baumgarten N, Schulz MH, Ebert P. TEPIC 2—an extended framework for transcription factor binding prediction and integrative epigenomic analysis. Bioinformatics 2018 10;https://dx.doi.org/10.1093/bioinformatics/bty856.
20. Trapnell C, Cacchiarelli D, Grimsby J, Pokharel P, Li S, Morse M, et al. The dynamics and regulators of cell fate decisions are revealed by pseudotemporal ordering of single cells. Nature Biotechnology 2014 03;32:381–386.
21. Li WV, Li JJ. An accurate and robust imputation method scImpute for single-cell RNA-seq data. Nature Communications 2018;9(1):997. https://doi.org/10.1038/s41467-018-03405-7.
22. Trapnell C, Cacchiarelli D, Monocle: Differential expression and time-series analysis for single-cell RNA-Seq and qPCR experiments; 2014. http://monocle-bio.sourceforge.net/monocle-vignette.pdf.
23. Friedman J, Hastie T, Tibshirani R. Regularization Paths for Generalized Linear Models via Coordinate Descent. Journal of Statistical Software 2010;33(1):1–22. http://www.jstatsoft.org/v33/i01/.
24. Kim S, Xing EP. Tree-Guided Group Lasso for Multi-Task Regression with Structured Sparsity. In: Proceedings of the 27th International Conference on Machine Learning (ICML-10), June 21-24, 2010, Haifa, Israel; 2010. p. 543–550. https://icml.cc/Conferences/2010/papers/352.pdf.
25. Gong W, Kwak IY, Pota P, Koyano-Nakagawa N, Garry DJ. DrImpute: imputing dropout events in single cell RNA sequencing data. BMC Bioinformatics 2018;19(1):220. https://doi.org/10.1186/s12859-018-2226-y.
26. Tracy S, Yuan GC, Dries R. RESCUE: imputing dropout events in single-cell RNA-sequencing data. BMC Bioinformatics 2019;20(1):388. https://doi.org/10.1186/s12859-019-2977-0.
27. Nguyen N, Zhang X, Olashaw N, Seto E. Molecular Cloning and Functional Characterization of the Transcription Factor YY2. Journal of Biological Chemistry 2004;279(24):25927–25934. http://www.jbc.org/content/279/24/25927.abstract.
28. Han H, Du Y, Zhao W, Li S, Chen D, Zhang J, et al. PBX3 is targeted by multiple miRNAs and is essential for liver tumour-initiating cells. Nature Communications 2015;6(1):8271. https://doi.org/10.1038/ncomms9271.
29. Liu T, Yang H, Fan W, Tu J, Li TWH, Wang J, et al. Mechanisms of MAFG Dysregulation in Cholestatic Liver Injury and Development of Liver Cancer. Gastroenterology 2018;155(2):557 – 571.e14. http://www.sciencedirect.com/science/article/pii/S0016508518344822.
30. Karyopherin α2-dependent import of E2F1 and TFDP1 maintains protumorigenic stathmin expression in liver cancer. Cell Communication and Signaling 2019;17(1):159. https://doi.org/10.1186/s12964-019-0456-x.
31. Rhee C, Lee BK, Beck S, Anjum A, Cook KR, Popowski M, et al. Arid3a is essential to execution of the first cell fate decision via direct embryonic and extraembryonic transcriptional regulation. Genes & Development 2014;28(20):2219–2232. http://genesdev.cshlp.org/content/28/20/2219.abstract.
32. Popowski M, Templeton TD, Lee BK, Rhee C, Li H, Miner C, et al. Bright/Arid3A Acts as a Barrier to Somatic Cell Reprogramming through Direct Regulation of Oct4, Sox2, and Nanog. Stem Cell Reports 2014;2(1):26 – 35. http://www.sciencedirect.com/science/article/pii/S2213671113001525.
33. Zheng C, Zheng L, Yoo JK, Guo H, Zhang Y, Guo X, et al. Landscape of Infiltrating T Cells in Liver Cancer Revealed by Single-Cell Sequencing. Cell 2017;169(7):1342 – 1356.e16. http://www.sciencedirect.com/science/article/pii/S0092867417305962.
34. Liu L, Liu C, Quintero A, Wu L, Yuan Y, Wang M, et al. Deconvolution of single-cell multi-omics layers reveals regulatory heterogeneity. Nature Communications 2019;10(1):470. https://doi.org/10.1038/s41467-018-08205-7.
35. Zhu C, Yu M, Huang H, Juric I, Abnousi A, Hu R, et al. An ultra high-throughput method for single-cell joint analysis of open chromatin and transcriptome. Nature Structural & Molecular Biology 2019;26(11):1063–1070. https://doi.org/10.1038/s41594-019-0323-x.
36. Clark SJ, Argelaguet R, Kapourani CA, Stubbs TM, Lee HJ, Alda-Catalinas C, et al. scNMT-seq enables joint profiling of chromatin accessibility DNA methylation and transcription in single cells. Nature Communications 2018;9(1):781. https://doi.org/10.1038/s41467-018-03149-4.

Click here to access/download
Supplementary Material
Triangulate_supplement.pdf

Personal Cover
Click here to access/download;Personal Cover;CoverLetter_resubmission.pdf

Cluster of Excellence
Multimodal Computing and Interaction
Saarland University &
Max Planck Institute for Informatics
German Center for Cardiovascular Research &
Uniklinikum and Goethe University &
Dr. Marcel H. Schulz
marcel.schulz@em.uni-frankfurt.de

Dear editor,

Please consider our revised manuscript GIGA-D-20-00071, titled as: *" Tree-based multi-tasking to predict gene regulation in single cells"* for publication in GigaScience.

We appreciate the effort the editor and reviewer have put in evaluating and criticizing our manuscript. We have addressed all the comments given by the reviewer and incorporated the requested changes to the text and included a point-by-point response to the reviewer's comments. Changes to the text are marked in cadmium green in the manuscript.

As part of our response to one of the reviewers, we acquired an additional data set for a single cell RNA-seq experiment, with details described in the main and supplemental text.
Also, we improved the documentation for the pipeline, as asked by one of the reviewers.

Our main findings remain unchanged: *TRIANGULATE* is a suitable tool for inferring transcription factor activity using single cell gene expression data and it is able to outperform its competing tool SCENIC, as shown on two data sets.

Thank you again for consideration of our revised manuscript. We are looking forward to your response.

Kind regards,

Prof. Dr. Marcel Schulz

Frankfurt am Main, 19.08.2020
