# Supplementary material for: Prediction of single-cell gene expression for transcription factor analysis
Source: Gigascience. 2020 Oct 30;9(11):giaa113. doi: 10.1093/gigascience/giaa113 (PMC7596801; doi:10.1093/gigascience/giaa113)
Supplement: giaa113_Supplemental_File [file giaa113_supplemental_file.pdf]

Placeholder for OUP logo oup.pdf

GigaScience, 2020, 1–6

doi: xx.xxxx/xxxx
Manuscript in Preparation
Paper

PAPER

Prediction of single cell gene expression for transcription factor analysis

Fatemeh Behjati Ardakani[1,2,3,4*], Kathrin Kattler[5], Tobias Heinen[2,3], Florian Schmidt[1,2,3,4], David Feuerborn[6], Gilles Gasparoni[5], Konstantin Lepikhov[5], Patrick Nell[6], Jan Hengstler[6], Jörn Walter[5] and Marcel H. Schulz[1,2,3*]

[1]Institute for Cardiovascular Regeneration, Goethe University, 60590 Frankfurt am Main, Germany and [2]Cluster of Excellence MMCI, Saarland University, Saarland Informatics Campus, 66123 Saarbrücken, Germany and [3]Max Planck Institute for Informatics, Saarland Informatics Campus, 66123 Saarbrücken, Germany and [4]Graduate School of Computer Science, Saarland University, Saarbrücken, Germany and [5]Department of Genetics, Saarland University, 66123 Saarbrücken, Germany and [6]Leibniz Research Centre for Working Environment and Human Factors (IfADo), 44139 Dortmund, Germany

*fbehjati@med.uni-frankfurt.de, +49 69 6301 86208; marcel.schulz@em.uni-frankfurt.de, +49 69 6301 86203

Supplemental methods

Generation and processing of HLC/PHH scRNA-seq data

We obtained single cell data for the study of Hepatocyte-like cells (HLC) that were *in vitro* differentiated either using the Cellartis iPS Cell to Hepatocyte Differentiation System (Cellartis, Takara Bio Europe AG, "CEL") or as described by [1]. Cryopreserved PHH were obtained from BioIVT. RNA-seq libraries of manually isolated single cells were generated using the Smart-seq2 protocol [2] and sequenced on a HiSeq2500 (Illumina) using TruSeq SBS Kit v3 – HS Chemistry in single read runs with 94 bp read length.

Reads were trimmed using Trim Galore! (http://www.bioinformatics.babraham.ac.uk/projects/trim_galore/)(v0.4.2) to remove 3′ ends with base quality below 20 as well as adapter sequences, and aligned to the human genome reference GRCh38 with STAR [3] with per sample 2-pass mapping strategy. PCR duplicates were detected using MarkDuplicate from Picard tools (http://broadinstitute.github.io/picard/ version 1.115). Gene counts were estimated based on Gencode release 30 (GRCh38.p12) using RSEM [4].

Processing of T cell scRNA-seq data

We utilized another single cell data set for our analysis. The data was measured for single cell expression in different cell types of patients with liver cancer. We obtained the read count data for patient P1116 (accession number GSM2602298) [5] to compare the top active TFs obtained from *TRIANGULATE* and SCENIC. We used the cells that are annotated as CD4+ cell type in normal (NTH, NTR) and tumor samples (TTH, TTR). We summarized the annotation of tumor cells (TTH, TTR) into *tumor* and normal cells (NTH, NTR) into *normal* for the analysis of TF regulation in these two types of T cells.

Training multi-task neural networks

Using the *keras* library, we trained a classic neural network on the filtered *static* features of the HLC/PHH and HSMM data sets. This network consists of six fully connected hidden layers, with 128, 128, 64, 64, 32 and 32 neurons. The input layer has as many nodes as the number of features. The number of nodes in the output layer is equal to the number of cells accounting for the multivariate response matrix of single cell data. The activation function used for all layers was *tanh*. We penalized the weight parameters by adding the L_1 and L_2 regularizations using the value of 0.00001 for both *l1_lambda* and *l2_lambda* hyper-parameters. We, also, used the *drop_out_rate* of 0.05 for all layers, except the input layer that was set to 0.1. The *batch_size* hyper-parameter was set to 32. The model was trained using 500 epochs accompanied by the early stopping option with *patience* value of 20. We complied the model with this architecture using the *mse* loss and *optimizer_rmsprop* optimizer.

Supplemental table and figures

Table 1. List of known TFs that are involved in regulating T cells.

T cell-specific TF	Related literature
YY1	[6]
ETV3	[6]
ETS1	[6]
ETV6	[6]
ATF1	[6]
NRF1	[6]
ZBTB33	[6]
IRF1	[6]
STAT2::STAT1	[6]
ZFP281	[6]
ZFX	[6]
ELF1	[6]
ZNF143	[6]
HSF1	[6]
TBP	[6]
FOXK1	[6]
GATA3	[6]
RUNX2	[6]
GABPA	[6]
BCL6	[6]
POU6f1	[6]
ZEB1	[6]
GFI1	[6]
JUN	[6]
SP4	[6]
CTCF	[6]
ZBTB3	[6]
ARID3A	[6]
RFX1	[6]
REST	[6]
ZBTB7B	[6]
ZFP691	[6]
SP100	[6]
GMEB1	[6]
USF2	[6]
BACH1::MAFK	[6]
SREBF1	[6]
MAFF	[6]
JUN::FOS	[6]
RUNX1	[6]
FOXP1	[6]
KLF5	[6]
SREBF2	[6]
NFKB1	[6]
ZFP187	[6]
SRF	[6]
NR3C1	[6]
MTF1	[6]
EWSR1-FLI1	[6]
ZFP161	[6]
RXRA	[6]
STAT6	[6]
NFATC2	[6]
POU2F2	[6]
FOXN2	[6]
USF1	[6]
ZNF354C	[6]
THAP1	[6]
ARID5A	[6]
CIC	[7]
MYB	[8]
ZNF70	[9]
HES1	[9]
KLF10	[10]
HOXA1	[11]
TCFL5	[11]
EGR3	[12]
NFATC1	[13]
NFYC	[14]
IRF9	[15]
E2F4	[16]
RELA	[17]
HIF1A	[18]

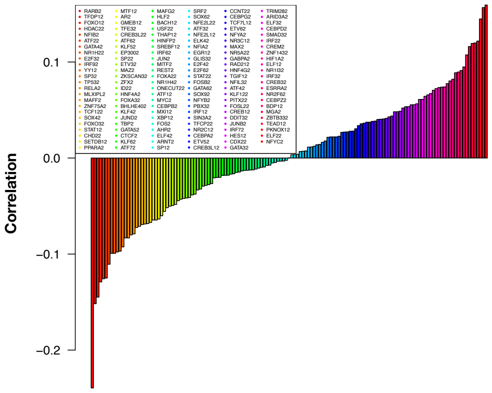

Figure 1. Spearman correlation between a TF's gene expression and inferred TF activity from the *R-tree* model over all cells in the HLC/PHH data set. These correlation values define the range of a null model that we used to discard TFs with low correlation values. TFs are sorted by correlation.

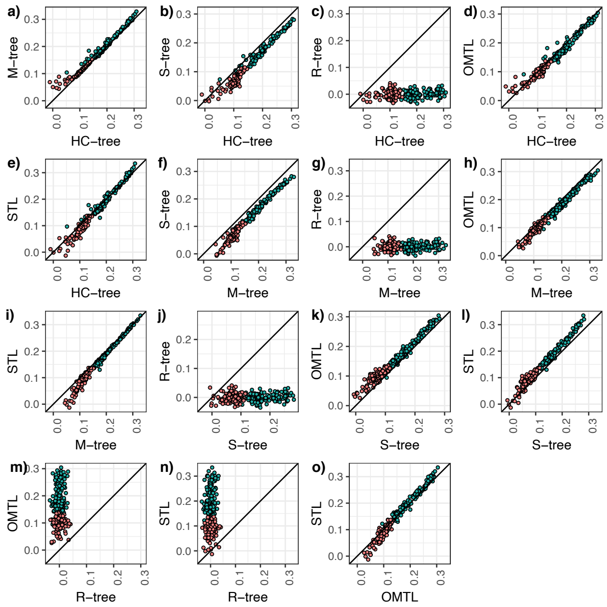

Figure 2. Each point in the scatter plot represents the Pearson correlation coefficient computed on the predicted and measured values of gene expression per cell (HLC/PHH data) for our statistical models (*HC-tree*, *M-tree*, *S-tree*, *R-tree*, *OMTL*, and *STL*). Correlation was computed using genes that are part of the test set. The diagonal line indicates the identity line to ease the comparison between the models placed on x and y axes. Circles colored by red and blue represent PHH and HLC cells, respectively.

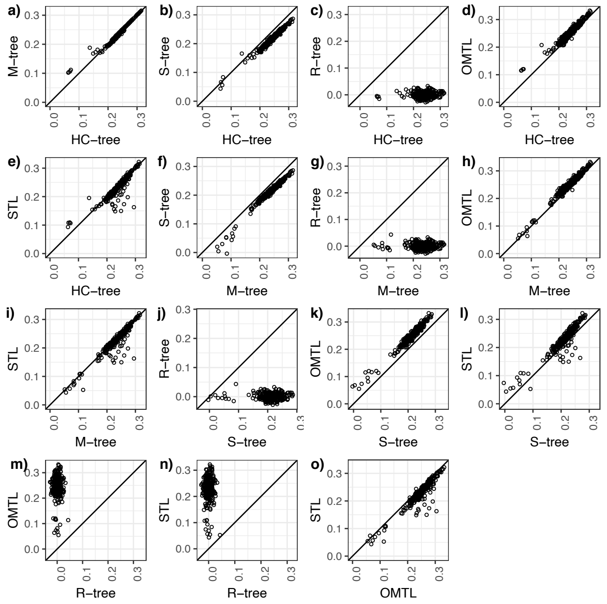

Figure 3. Each point in the scatter plot represents the Pearson correlation coefficient computed between the predicted and measured values of gene expression per cell (HSMM) for our statistical models (*HC-tree*, *M-tree*, *S-tree*, *R-tree*, *OMTL*, and *STL*). Correlation was computed using genes that are part of the test set. The diagonal line indicates the identity line to ease the comparison between the models placed on x and y axes.

a) **HSMM**

	HC−tree	M−tree	S−tree	R−tree	OMTL	STL
HC−tree	1	0.264	0	0	1e−04	0.0012
M−tree	0.264	1	0	0	0	0.0437
S−tree	0	0	1	0	0	0
R−tree	0	0	0	1	0	0
OMTL	1e−04	0	0	0	1	0
STL	0.0012	0.0437	0	0	0	1

b) **HLC/PHH**

	HC−tree	M−tree	S−tree	R−tree	OMTL	STL
HC−tree	1	0.2608	0	0	0.0824	0.0738
M−tree	0.2608	1	0	0	0.0057	0.0056
S−tree	0	0	1	0	1e−04	5e−04
R−tree	0	0	0	1	0	0
OMTL	0.0824	0.0057	1e−04	0	1	0.7627
STL	0.0738	0.0056	5e−04	0	0.7627	1

Figure 4. Comparison of statistical models on test data of static features, for HSMM (a) and HLC/PHH (b) data sets. Two sided Mann-Whitney (unpaired) test with significance cutoff of 0.05 was performed on the prediction correlation values.

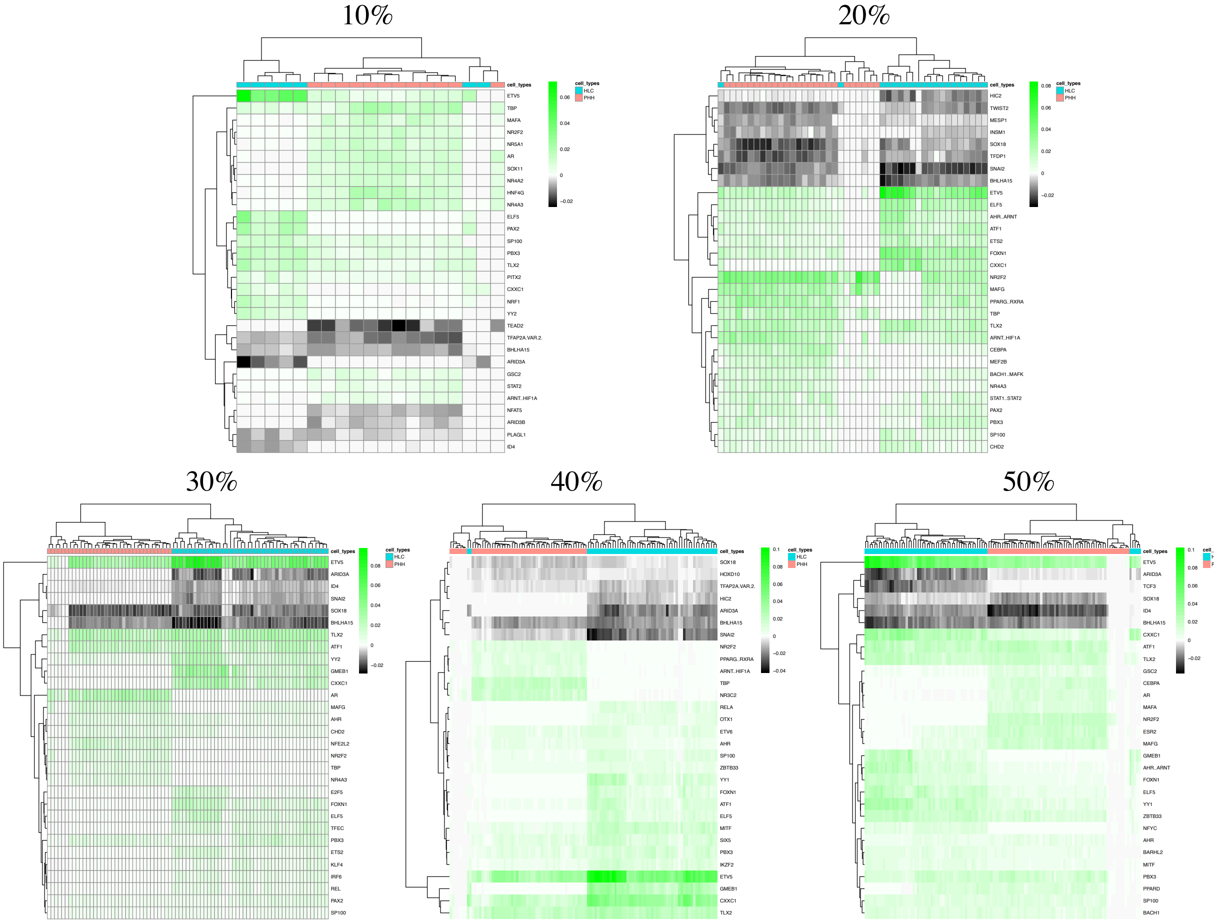

Figure 5. Heat maps showing the *TRIANGULATE* model coefficients trained on static features with down-sampled cells (10%–50% of the total cell population). The cell annotation (columns) show a clear separation of the two cell types based on these coefficient values.

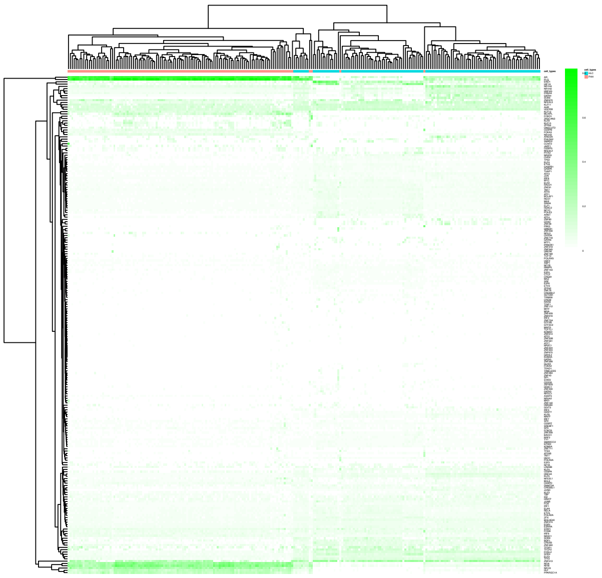

Figure 6. Heat map illustrating SCENIC AUC values obtained on the HLC/PHH data. Rows are TFs that SCENIC used in its analysis and columns are the cells clustered by heat map's built-in clustering. White color in the heat map represents AUC value of 0 and as the AUC values increase the green color intensity increases as well.

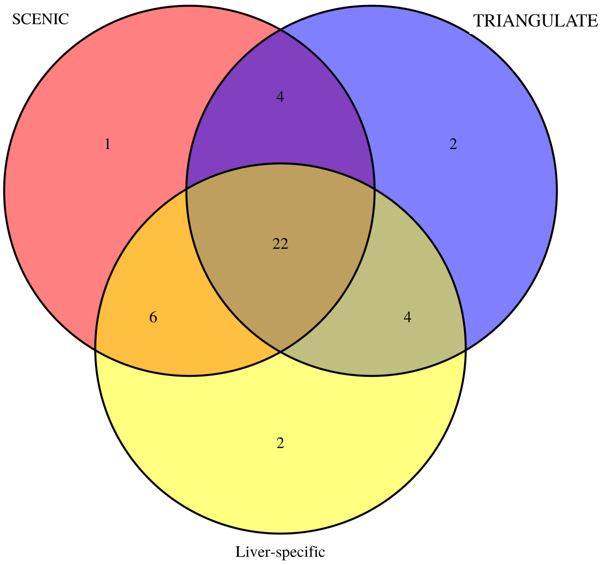

Figure 7. Venn diagram depicting the number of overlapping TFs that are significantly distinct between the PHH and HLC cell types (two-sided Mann-Whitney test using the significance cutoff of 0.1 on the multiple-testing corrected p-values (Benjamini-Hochberg method)). Tree-guided MTL denoted as TRIANGULATE. Liver-specifc denotes a set of TFs that were obtained by literature search of known liver-specifc TFs [6].

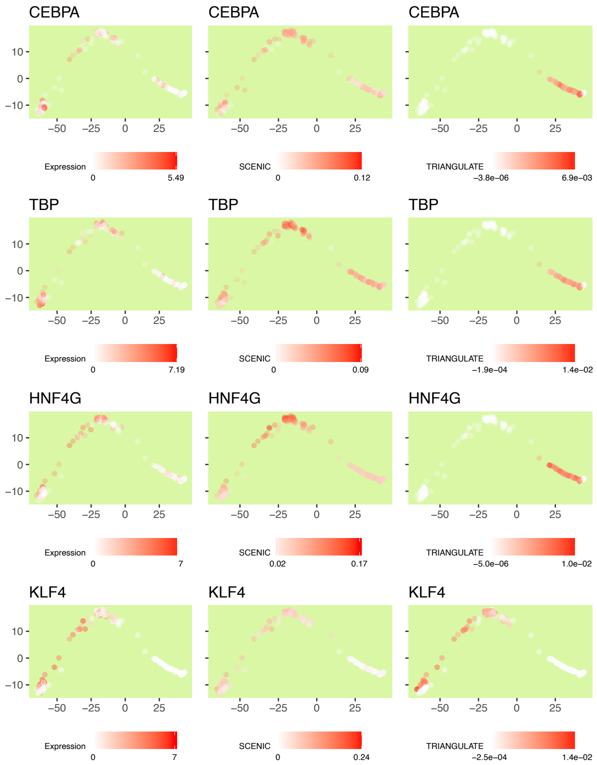

Figure 8. Cells arranged according to the 2D trajectory obtained from Monocle and colored based on the inferred TF activity (SCENIC or TRIANGULATE) for the liver-specific TFs that were detected only by TRIANGULATE and not SCENIC (CEBPA, TBP, HNF4G, and KLF4). The expression of each TF is also plotted in the single cell trajectory. The darker the color, the stronger the activity.

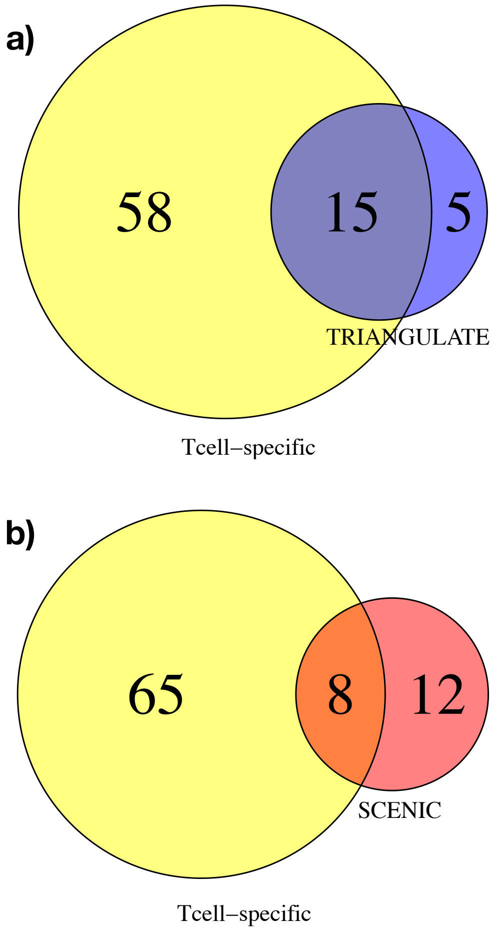

Figure 9. Venn diagrams depicting the overlap between the set of T cell-specifi TFs and the top 20 active TFs obtained from (a) *TRIANGULATE* and (b) SCENIC, for the T cell data sets.

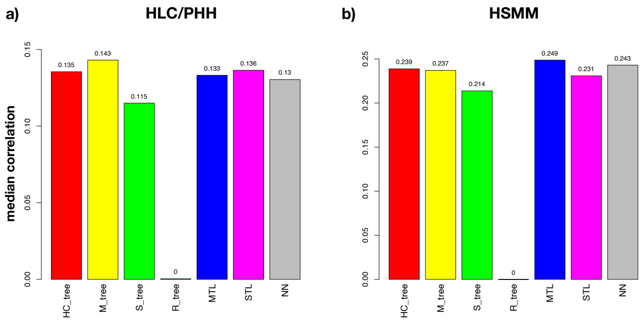

Figure 10. Median of the test correlation values obtained across cells. This includes all types of statistical models (including the MLP neural network (NN, indicated by gray color)) on the static features and unimputed single cell RNA-seq, for HLC/PHH (a) and HSMM (b) data sets.

References

1. AU Wang Y, AU Alhaque S, AU Cameron K, AU Meseguer-Ripolles J, AU Lucendo-Villarin B, AU Rashidi H, et al. Defined and Scalable Generation of Hepatocyte-like Cells from Human Pluripotent Stem Cells. JoVE 2017;e55355(121). https://www.jove.com/video/55355.
2. Picelli S, Björklund ÅK, Faridani OR, Sagasser S, Winberg G, Sandberg R. Smart-seq2 for sensitive full-length transcriptome profiling in single cells. Nature Methods 2013;10(11):1096–1098. https://doi.org/10.1038/nmeth.2639.
3. Dobin A, Davis CA, Schlesinger F, Drenkow J, Zaleski C, Jha S, et al. STAR: ultrafast universal RNA-seq aligner. Bioinformatics 2012;29(1):15–21. https://doi.org/10.1093/bioinformatics/bts635.
4. Li B, Dewey CN. RSEM: accurate transcript quantification from RNA-Seq data with or without a reference genome. BMC Bioinformatics 2011;12(1):323. https://doi.org/10.1186/1471-2105-12-323.
5. Zheng C, Zheng L, Yoo JK, Guo H, Zhang Y, Guo X, et al. Landscape of Infiltrating T Cells in Liver Cancer Revealed by Single-Cell Sequencing. Cell 2017;169(7):1342 – 1356.e16. http://www.sciencedirect.com/science/article/pii/S0092867417305962.
6. Schmidt F, Gasparoni N, Gasparoni G, Gianmoena K, Cadenas C, Polansky JK, et al. Combining transcription factor binding affinities with open-chromatin data for accurate gene expression prediction. Nucleic acids research 2017;45(1):54–66.
7. Tan Q, Brunetti L, Rousseaux MWC, Lu HC, Wan YW, Revelli JP, et al. Loss of Capicua alters early T cell development and predisposes mice to T cell lymphoblastic leukemia/lymphoma. Proceedings of the National Academy of Sciences 2018;115(7):E1511–E1519. https://www.pnas.org/content/115/7/E1511.
8. Gautam S, Fioravanti J, Zhu W, Le Gall JB, Brohawn P, Lacey NE, et al. The transcription factor c-Myb regulates CD8+ T cell stemness and antitumor immunity. Nature Immunology 2019;20(3):337–349. https://doi.org/10.1038/s41590-018-0311-z.
9. Watanabe K, Kazuhiro N, Ohta S, Tago K, Boonvisut S, Millings E, et al. ZNF70, a novel ILDR2-interacting protein, contributes to the regulation of HES1 gene expression. Biochemical and Biophysical Research Communications 2016;477.
10. Khedkar SA, Sun X, Rigby AC, Feinberg MW. Discovery of Small Molecule Inhibitors to Krüppel-like Factor 10 (KLF10): Implications for Modulation of T Regulatory Cell Differentiation. Journal of Medicinal Chemistry 2015;58(3):1466–1478. https://doi.org/10.1021/jm5018187.
11. Weerkamp F, Luis TC, Naber BAE, Koster EEL, Jeannotte L, van Dongen JJM, et al. Identification of Notch target genes in uncommitted T-cell progenitors: no direct induction of a T-cell specific gene program. Leukemia 2006;20(11):1967–1977. https://doi.org/10.1038/sj.leu.2404396.
12. Li S, Miao T, Sebastian M, Bhullar P, Ghaffari E, Liu M, et al. The transcription factors Egr2 and Egr3 are essential for the control of inflammation and antigen-induced proliferation of B and T cells. Immunity 2012;37(4):685–696. https://pubmed.ncbi.nlm.nih.gov/23021953, 23021953[pmid].
13. Klein-Hessling S, Muhammad K, Klein M, Pusch T, Rudolf R, Flöter J, et al. NFATc1 controls the cytotoxicity of CD8(+) T cells. Nature communications 2017;8(1):511–511. https://pubmed.ncbi.nlm.nih.gov/28894104, 28894104[pmid].
14. Takenoyama M, Baurain JF, Yasuda M, So T, Sugaya M, Hanagiri T, et al. A point mutation in the NFYC gene generates an antigenic peptide recognized by autologous cytolytic T lymphocytes on a human squamous cell lung carcinoma. International Journal of Cancer 2006;118(8):1992–1997. https://onlinelibrary.wiley.com/doi/abs/10.1002/ijc.21594.
15. Huber M, Suprunenko T, Ashhurst T, Marbach F, Raifer H, Wolff S, et al. IRF9 Prevents CD8+ T Cell Exhaustion in an Extrinsic Manner during Acute Lymphocytic Choriomeningitis Virus Infection. Journal of Virology 2017;91(22). https://jvi.asm.org/content/91/22/e01219-17.
16. Bancos S, Cao Q, Bowers WJ, Crispe IN. Dysfunctional memory CD8+ T cells after priming in the absence of the cell cycle regulator E2F4. Cellular immunology 2009;257(1-2):44–54. https://pubmed.ncbi.nlm.nih.gov/19306992, 19306992[pmid].
17. Ronin E, Lubrano di Ricco M, Vallion R, Divoux J, Kwon HK, Grégoire S, et al. The NF-B RelA Transcription Factor Is Critical for Regulatory T Cell Activation and Stability. Frontiers in Immunology 2019;10:2487. https://www.frontiersin.org/article/10.3389/fimmu.2019.02487.
18. Cho SH, Raybuck AL, Blagih J, Kemboi E, Haase VH, Jones RG, et al. Hypoxia-inducible factors in CD4+ T cells promote metabolism, switch cytokine secretion, and T cell help in humoral immunity. Proceedings of the National Academy of Sciences 2019;116(18):8975–8984. https://www.pnas.org/content/116/18/8975.
