# Supplementary material for: Prediction of single-cell gene expression for transcription factor analysis
Source: Gigascience. 2020 Oct 30;9(11):giaa113. doi: 10.1093/gigascience/giaa113 (PMC7596801; doi:10.1093/gigascience/giaa113)
Supplement: giaa113_Response_to_Reviewer_Comments_Original_Submission [file giaa113_response_to_reviewer_comments_original_submission.pdf]

Author's Response To Reviewer Comments

Close

Reviewer reports:
Reviewer #1: In the manuscript, Ardakani et.al. described a novel computational framework that can predict gene expression level at single cell level based on scRNA-seq data as well as the inferred TF activity. They demonstrated its superior performance by applying it in two datasets. Overall, the manuscript is well written except a couple of places with minor grammar errors. I believe the tool developed by them will be of interest to the single-cell genomics community. I have only two minor comments/suggestions.
###
1. In fig4a. it is unclear whether the Tree-guided outperforms OMTL and STL. How would OMTL compare to STL? In Fig4b, visually the difference between HC-tree band OMTL is not that big, how would M-tree compare to OMTL? How would S-tree compare to OMTL and STL?

We apologize for not providing the statistics on all pairs, in the first version of the manuscript. We now added figures to our supplemental materials that provide the Mann-Whitney test p-values on all pairs of models (Supp. Fig. 3).

###
2. The author should discuss how the performance of their tool would be potentially affected by the features of scRNA-seq datasets, e.g. cell numbers, number of cells in different clusters, No. UMI/cell,No. genes/cell and etc.

This is a good idea, thank you. We added a new subsection in the Results section of the manuscript (The impact of the number of cells on prediction results) on the use of TRIANGULATE on single cell datasets of different scale and quality.

Reviewer #2: In this manuscript the authors developed a workflow to identify transcription factors (TFs) that may have cell type-specific regulation from single-cell RNA-seq (scRNA-seq) data. To achieve this, they tested many different methods: to generate proper TF feature matrices they use static, dynamic and ChIP-seq features; for statistical learning frameworks they use single- and multi-task learning methods. Furthermore, they generated several trees to guide the optimization of the tree-guided MTL models. By systematic comparisons, they found HC-tree and ChIP-seq showed better performing results in the data sets they tested. And finally, they compared the results with SCENIC, which is one of the most commonly used software to explore gene regulatory networks within scRNA-seq data. They found that TRIANGULATE had better performance in TF motif prediction and more important, TRIANGULATE can predict negatively correlated TF-gene pairs, which is a unique feature to TRIANGULATE. This manuscript is suitable for GigaScience, however, some issues need to be addressed before it can be considered for publication.
###
1. The authors tested only two cell types (HLC and PHH), can TRIANGULATE be applied to other data with similar performance as well? In other words, in addition to HLC and PHH, how is the performance of TRIANGULATE compared to SCENIC?

Thanks for raising this point. To address that, we gathered T cells from a human HCC patient (GSM2602298) with normal and tumor cell annotations. We ran TRIANGULATE and SCENIC on this data, and the results are described in the Results section (Comparison of results with SCENIC subsection) of the manuscript.
In brief, our method performs similar to the HLC/PHH dataset in terms of prediction accuracy measured in Pearson correlation. To compare it with SCENIC and a set of known T cell specific TFs, we overlapped the set of known T cell specific TFs with the set of top 20 active TFs from TRIANGULATE and SCENIC, where we found 15 and 8 hits, respectively.

###

2. The title seems too broad and it is hard to get the main point of this manuscript.

We are sorry that the manuscript's title did not seem very right to the point, but we decided to choose the "Prediction of single cell gene expression for transcription factor analysis" title for the following reasons:
- A predictive model is proposed that predicts the single cell gene expression, therefore "Prediction of single cell gene expression"
- This model enables us to infer and analyze the transcription factor activity within these single cells, therefore "for transcription factor analysis".
Thus, we think that the title reflects the core contribution of our manuscript.
###
3. The authors used 2 kb to define the promoter of a certain gene. How is this threshold selected? This is important because it affects the performance of TRIANGULATE.

The threshold is selected on the basis of previous work with sequence based motif prediction in the context of gene regulation, where we found that including motif hits in a window larger than 2kb is not working well.
###
4. It would be better to import the matrix directly instead of using Monocle, because the users may have their preferred methods to filter their data.

Yes, that is a valid point. We added the option to directly import the matrix to Triangulate. The details are added to the GitHub readme page.
###
5. The interface and description of TRIANGULATE in GitHub are simple. The authors should provide a more detailed tutorial and add more annotations, such as the meaning of the functions and variables listed on the website.

We are sorry that the description was insufficient. We improved the description of the scripts, their arguments, and variable names, as well as the purpose of those scripts as part of the snakemake workflow rules.

Close
