# Supplementary material for: Prediction of single-cell gene expression for transcription factor analysis
Source: Gigascience. 2020 Oct 30;9(11):giaa113. doi: 10.1093/gigascience/giaa113 (PMC7596801; doi:10.1093/gigascience/giaa113)
Supplement: giaa113_Reviewer_2_Report_Original_Submission — Longqi Liu -- 5/23/2020 Reviewed [file giaa113_reviewer_2_report_original_submission.pdf]

Reviewer Report

Title: Prediction of single cell gene expression for transcription factor analysis

Version: Original Submission **Date:** 5/23/2020

Reviewer name: Longqi Liu

Reviewer Comments to Author:

In this manuscript the authors developed a workflow to identify transcription factors (TFs) that may have cell type-specific regulation from single-cell RNA-seq (scRNA-seq) data. To achieve this, they tested many different methods: to generate proper TF feature matrices they use static, dynamic and ChIP-seq features; for statistical learning frameworks they use single- and multi-task learning methods. Furthermore, they generated several trees to guide the optimization of the tree-guided MTL models. By systematic comparisons, they found HC-tree and ChIP-seq showed better performing results in the data sets they tested. And finally, they compared the results with SCENIC, which is one of the most commonly used software to explore gene regulatory networks within scRNA-seq data. They found that TRIANGULATE had better performance in TF motif prediction and more important, TRIANGULATE can predict negatively correlated TF-gene pairs, which is a unique feature to TRIANGULATE. This manuscript is suitable for GigaScience, however, some issues need to be addressed before it can be considered for publication.

1. The authors tested only two cell types (HLC and PHH), can TRIANGULATE be applied to other data with similar performance as well? In other words, in addition to HLC and PHH, how is the performance of TRIANGULATE compared to SCENIC?
2. The title seems too broad and it is hard to get the main point of this manuscript.
3. The authors used 2 kb to define promoter of a certain gene. How is this threshold selected? This is important because it affects the performance of TRIANGULATE.
4. It would be better to import the matrix directly instead of using Monocle, because the users may have their preferred methods to filter their data.
5. The interface and description of TRIANGULATE in GitHub are simple. The authors should provide a more detailed tutorial and add more annotations, such as the meaning of the functions and variables listed on the website.

Methods

Are the methods appropriate to the aims of the study, are they well described, and are necessary controls included? Choose an item.

Conclusions

Are the conclusions adequately supported by the data shown? Choose an item.

Reporting Standards

Does the manuscript adhere to the journal's guidelines on minimum standards of reporting? Choose an item.

Choose an item.

Statistics

Are you able to assess all statistics in the manuscript, including the appropriateness of statistical tests used? Choose an item.

Quality of Written English

Please indicate the quality of language in the manuscript: Choose an item.

Declaration of Competing Interests

Please complete a declaration of competing interests, considering the following questions:

- Have you in the past five years received reimbursements, fees, funding, or salary from an organisation that may in any way gain or lose financially from the publication of this manuscript, either now or in the future?
- Do you hold any stocks or shares in an organisation that may in any way gain or lose financially from the publication of this manuscript, either now or in the future?
- Do you hold or are you currently applying for any patents relating to the content of the manuscript?
- Have you received reimbursements, fees, funding, or salary from an organization that holds or has applied for patents relating to the content of the manuscript?
- Do you have any other financial competing interests?
- Do you have any non-financial competing interests in relation to this paper?

If you can answer no to all of the above, write 'I declare that I have no competing interests' below. If your reply is yes to any, please give details below.

I declare that I have no competing interests

I agree to the open peer review policy of the journal. I understand that my name will be included on my report to the authors and, if the manuscript is accepted for publication, my named report including any attachments I upload will be posted on the website along with the authors' responses. I agree for my report to be made available under an Open Access Creative Commons CC-BY license (http://creativecommons.org/licenses/by/4.0/). I understand that any comments which I do not wish to be included in my named report can be included as confidential comments to the editors, which will not be published.

Choose an item.

To further support our reviewers, we have joined with Publons, where you can gain additional credit to further highlight your hard work (see: https://publons.com/journal/530/gigascience). On publication of this paper, your review will be automatically added to Publons, you can then choose whether or not to claim your Publons credit. I understand this statement.

Yes Choose an item.
